# Misreporting Month of Birth: Diagnosis and Implications for Research on Nutrition and Early Childhood in Developing Countries

**DOI:** 10.1007/s13524-018-0753-9

**Published:** 2019-01-28

**Authors:** Anna Folke Larsen, Derek Headey, William A. Masters

**Affiliations:** 1 Department of Economics, University of Copenhagen, Harespringet 3, Copenhagen NV, Denmark; 2 Poverty, Health, and Nutrition Division, International Food Policy Research Institute, 1201 Eye Street, Washington, DC 20005-3915 USA; 3 Friedman School of Nutrition and Department of Economics, Tufts University, Boston, MA 02111 USA

**Keywords:** Nutrition, Height-for-age, Stunting, Measurement error, Child age

## Abstract

A large literature has used children’s birthdays to identify exposure to shocks and estimate their impacts on later outcomes. Using height-for-age *z* scores (HAZ) for more than 990,000 children in 62 countries from 163 Demographic and Health Surveys (DHS), we show how random errors in birth dates create artifacts in HAZ that can be used to diagnose the extent of age misreporting. The most important artifact is an upward gradient in HAZ by recorded month of birth (MOB) from start to end of calendar years, resulting in a large HAZ differential between December- and January-born children of –0.32 HAZ points. We observe a second artifact associated with round ages, with a downward gradient in HAZ by recorded age in months, and then an upward step after reaching ages 2, 3, and 4. These artifacts have previously been interpreted as actual health shocks. We show that they are not related to agroclimatic conditions but are instead linked to the type of calendar used and arise mainly when enumerators do not see the child’s birth registration cards. We explain the size of the December–January gap through simulation in which 11 % of children have their birth date replaced by a random month. We find a minor impact on the average stunting rate but a larger impact in specific error-prone surveys. We further show how misreporting MOB causes attenuation bias when MOB is used for identification of shock exposure as well as systematic bias in the impact on HAZ of events that occur early or late in each calendar year.

**Electronic supplementary material:**

The online version of this article (10.1007/s13524-018-0753-9) contains supplementary material, which is available to authorized users.

## Introduction

A large literature has investigated the impact of events in pregnancy and early childhood on later outcomes, including many studies in low-income countries (Currie and Vogl 2013; Rieger and Trommlerová 2016). This kind of research relies on accurate birth dates to measure each child’s exposure to a given shock and each child’s stature relative to healthy children of their age and sex. Low height-for-age is a particularly significant public health concern, given that approximately one-quarter of the world’s preschool-age population are classified as stunted (UNICEF 2015) with numerous consequences for health (Black et al. 2008, 2013), child development (Grantham-McGregor et al. 2007), and education and economic life (Dewey and Begum 2011; Hoddinott et al. 2013). Stunting—a preferred policy and program indicator for monitoring changes in undernutrition—is widely used as a development target, including for the Sustainable Development Goals and The World Health Assembly targets.

The significant attention given to early life shocks and to stunting makes it important to know whether birth dates are measured accurately and to understand any biases introduced by errors in existing data. Children are classified as *stunted* if their height (or length) for their age is low relative to the World Health Organization’s (WHO) worldwide reference population of healthy children (WHO 2006; de Onis et al. 2007). Differences are measured as *z* scores, in units of standard deviation relative to the mean height of healthy children at that age and sex. A height-for-age *z* score (HAZ) of less than –2 standard deviations (SD) is considered stunted, and an HAZ ≤ 3 SD is considered severely stunted.

The causes and consequences of errors in HAZ have received considerable attention (Mei and Grummer-Strawn 2007), including from researchers involved in the Demographic and Health Surveys (DHS) produced by ICF International (2015), such as Assaf et al. (2015). Studies of measurement have typically focused on height itself, and misreporting of child age has been little studied despite the difficulty of measuring exact ages and their importance for estimated HAZs and stunting rates. Misreporting of age could be especially important for analysis of month-to-month changes in early childhood, when most growth faltering occurs (Leroy et al. 2014; Shrimpton et al. 2001; Victora et al. 2010).

In any given survey, the exact birth date and actual age of children may be unknown because of low numeracy and literacy, lack of birth registration, and limited celebration of birthdays or regular use of conventional calendars. At the same time, statistical agencies rightly try to avoid the selection bias that would arise from omitting children whose age is uncertain (Croft 1991). Survey enumerators are strongly encouraged to work with respondents to identify plausible ages. In the DHS, enumerators are trained to elicit an age in years or a birth year for each child and to use salient events or seasons to narrow down toward the best available estimate of birth month (ICF-Macro 2009). Some DHS studies have nonresponse rates for child ages as high as 30 %, while other surveys in similar settings report birth years and months for 98 % to 99 % of respondents. Enumerators in the latter surveys clearly make considerable efforts to elicit a response and thereby limit selection bias, despite the resulting scope for either random or systematic misreporting.

Previous studies of age reporting in DHS data have focused on role of written birth records (Comandini et al. 2016), specific interview procedures (Assaf et al. 2015), and age thresholds (such as when children are falsely reported as older than 59 months) to avoid having to measure their heights and weights (Pullum 2006). Age or birth date recall data may also be checked for heaping at cognitive anchors, such as round numbers, using standard diagnostics such as Whipple’s or Myers’s indexes. For example, Assaf et al. (2015) used Myers’s index to show that no more than 10 % of children’s ages would need to be reallocated to eliminate age heaping. Such corrections, if made, would be symmetric displacements around the cognitive anchor.

In this study, we use DHS data from 62 countries between 1990 and 2014 to quantify an important anomaly in HAZ data and show how it could arise from random errors in recorded month of birth (MOB). The anomaly we observe is an upward gradient in HAZ from start to end of calendar years, with a downward step from December to January of 0.32 HAZ points. We show how that asymmetric sawtooth pattern can readily arise by random misreporting of months within years. We term this pattern the *calendar-year artifact*. Random errors in MOB produce a nonrandom pattern of HAZ because children who are mistakenly reported as born later in the year were actually born earlier and therefore appear taller for their age than they actually are, and vice versa for those mistakenly reported as born earlier in the year. We also observe another sawtooth pattern around age in completed years, with a downward gradient in HAZ by recorded age in months, and then an upward step after reaching the round ages 2, 3, and 4 years. We call this the *round-age artifact*. To our knowledge, our study is the first to document the round-age artifact. These two artifacts are not confined to DHS data, and we find similar patterns in other surveys.

The calendar-year artifact has been previously observed, most recently by Agarwal et al. (2017), but has been attributed to unknown causes or seasonality in environmental influences on health (Dorelien 2015; Lokshin and Radyakin 2012). By combining data from diverse geographic regions, we show that the artifacts are not related to agroclimatic conditions but are linked to the type of calendar used, and that they arise mainly when enumerators do not see the child’s birth registration cards. Using a simulation model, we estimate the magnitude of random error needed to replicate the anomalies observed in DHS data, the impact of misreporting on measured stunting rates, and the biases they introduce when estimating the effect of an early-life shock on attained height.

We find that the December–January gap of –0.32 HAZ points can be explained by replacing the birth month with a random month for 11 % of the children. Although we find only a minor impact on the average stunting rate across the 62 countries studied, we demonstrate that impacts may be larger for specific surveys or subgroups that are more error-prone than average. Furthermore, we show how misreporting MOB causes not just attenuation bias but also sizable systematic bias in the impact on HAZ of events that occur early or late in each calendar year.

## Anomalies in Global HAZ Data

**Fig. 1:**
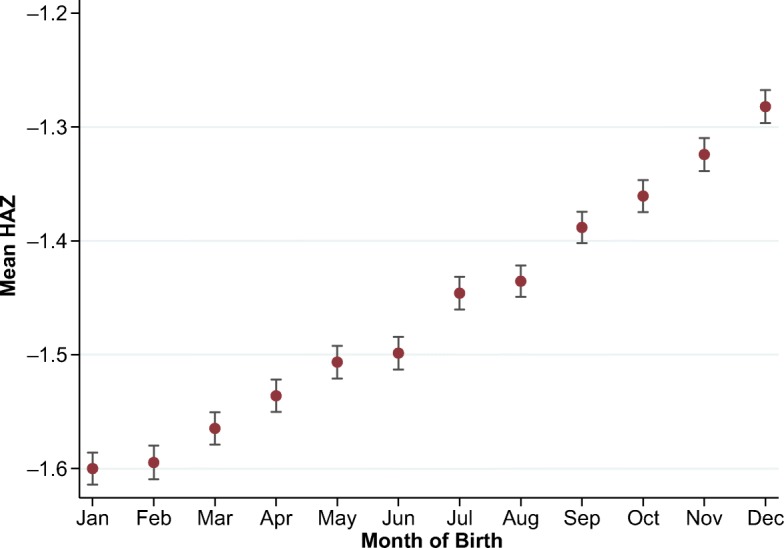
Mean height-for-age *z* scores (HAZ) by calendar month of birth (MOB). The vertical bars indicate standard errors of the mean HAZ. *Source:* DHS data for 990,231 children from 62 countries, various years.

The principal motivation for this study is the anomaly in mean HAZ scores by calendar month of birth shown in Fig. 1. This chart pools the multicountry data on attained HAZ for all children ever measured in the DHS. Sample size by region is described in Table A1 in the online appendix. In total, this sample includes just under 1 million children from 163 surveys covering 62 countries. Almost one-half the children are from 33 countries in sub-Saharan Africa; 10 % are from the five countries of South Asia; and the remainder are from Latin America, eastern Europe, East Asia and the Pacific, or the Middle East and North Africa.

The smooth upward gradient shown in Fig. 1 implies that each successive calendar month contributes equally to higher HAZ from January to December, followed by an implausibly large step down from the end of one year to the start of the next. For the DHS as a whole, Fig. 1 reveals that the overall December–January gap is approximately 0.32 HAZ points. This phenomenon has been reported previously for specific regions and attributed to local climatic or environmental factors. Lokshin and Radyakin (2012) found a rise in HAZs all across India with each calendar month, for a cumulative December–January gap of 0.37 SD. In those data, the December–January gap is equivalent to the HAZ difference between children whose mothers have no education at all and those whose mothers completed secondary education. Dorelien (2015) found a similar gradient across 30 African countries, with a December–January gap that sums to a difference in stunting prevalence of about 3 percentage points.^1^ When we pool worldwide data across both hemispheres and a wide range of environmental and socioeconomic conditions as in Fig. 1, we find a global pattern that cannot plausibly be explained by genuine seasonality in child outcomes, but no alternative explanation has yet been provided.

**Fig. 2:**
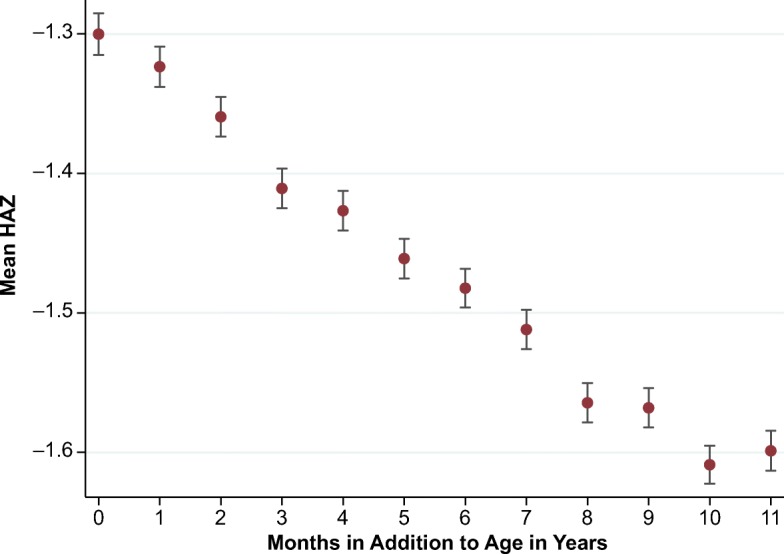
Mean height-for-age *z* scores (HAZ) by age in months after a round age. The vertical bars indicate standard errors of the mean HAZ. *Source:* DHS data for 990,231 children from 62 countries, various years.

**Fig. 3:**
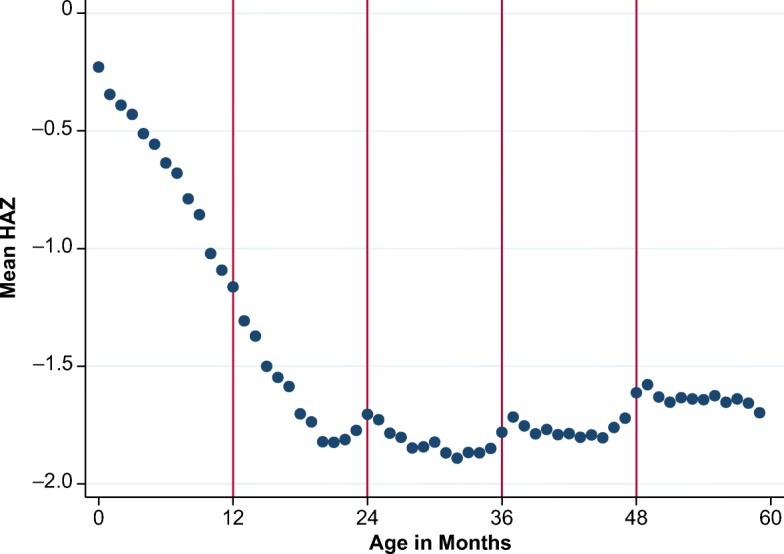
Mean height-for-age *z* scores (HAZ) by age in months. *Source:* DHS data for 990,231 children from 62 countries, various years.

A separate anomaly that has not been previously reported is a downward gradient in HAZ by months after completed years, followed by a discrete step up around each complete age in years, as shown in Fig. 2. This sawtooth pattern is more subtle than the calendar-year anomaly in Fig. 1, if only because it coincides with an actual decline in HAZ in the first two years after birth (Victora et al. 2010) and debates over the degree of recovery after age 2 (Leroy et al. 2014), as shown in Fig. 3.

Our principal focus in this article is the calendar-year anomaly of Fig. 1, in part because the resulting December–January gap provides a clearer diagnostic indicator than the round-age gap at ages 2, 3, and 4 that is visible in Figs. 2 and 3. We show that the two anomalies occur independently, meaning that the one anomaly does not lead to the other. If they result from misreporting, they would both create a misclassification problem for econometric analyses that rely on reported age to identify exposure to early-life shocks, policies, or programs. For example, Akresh et al. (2011) noted the possible attenuation bias caused by age errors, leading to underestimation of impacts on HAZ of exposure to conflict and drought in Rwanda. In general, misclassification of exposure will lead to attenuation bias in the estimated impact on any outcome variable (Aigner 1973). Errors in age could also cause directional biases—for example, by increasing the spread of the HAZ distribution and thereby raising estimates of stunting prevalence, or by introducing spurious correlation between specific birth months and HAZ outcomes that bias estimates of exposure to genuinely seasonal shocks.

The phenomena we describe could arise in any survey, but we confine our analysis to DHS data for several reasons. First, the DHS are the single largest source of nationally representative nutritional data in the developing world, widely used for nutrition monitoring and analysis by WHO, the Global Nutrition Report, and many other institutions and individual researchers. Second, the availability of DHS surveys for multiple countries allows us to draw comparisons across countries with different agroclimatic seasons, levels of education and birth documentation, and cultural norms. Third, the DHS surveys are relatively standardized, with enumerators receiving similar training on topics such as age measurement.

Our analysis addresses the relationship between height and age in all available DHS rounds that collected anthropometric indicators for children 0 to 59 months old (excluding surveys that sampled only children 0 to 36 months of age). Anthropometric data collection has been a key component of the DHS since 1986, focusing on the length (for children 0–24 months), height (25–59 months), and weight of children under age 5 who stayed in the household the night before the survey. Reducing error in anthropometry is a long-standing focus of DHS and other surveys, but the accurate recall of children’s age has received less attention and may actually have become more difficult over time because of the increasing length and complexity of each interview. For example, standard DHS phase III surveys administered in the 1990s included 247 questions, whereas DHS phase VII surveys used since 2013 have 425 questions.

## Survey Procedures and Age Misreporting

Before any further analysis of the data, it is helpful to consider how surveys actually elicit birth dates when written records are not available and how two sorts of random misreporting around a child’s true age could generate the systematic biases and artifacts shown in Figs. 1 and 2.

In the DHS, as in many surveys, data on a child’s age are obtained from a caregiver, usually the mother. DHS interviewers are typically national staff from private or government statistical agencies who receive extensive training on how to obtain and record height and weight measurements as well as the birth date. Additional measures include field check tables, multiple layers of supervision, and field visits as part of standard protocols informed by DHS research on data quality (Assaf et al. 2015; Pullum 2006).

To elicit age, enumerators ask respondents for the year and MOB of all children born in a household, living or dead, as well as the exact date (day) of birth. Surveys include the age at last birthday of all living children, although this is used only as a check on the calculated age in months, which is used for HAZ scores. When dates are not immediately recalled or available from written documents, enumerators are trained to start with completed age in years or the birth year for each child and then to elicit the birth month based on the caregiver’s recollection of memorable events around that time (ICF-Macro 2009). If no day in the month is recorded for birth, the DHS assigns the number 15. If no month is recorded, enumerators have the option of recording this as missing for later imputation, but this is rarely done. For example, in India, only 0.61 % of children have no birth month directly recorded by an enumerator; in Nigeria, just 1.36 % have no month recorded. Only Guinea (14.7 %), Benin (8.7 %), and Burkina Faso (5.6 %) have notable proportions of imputation on birth month. As shown in Fig. A1 in the online appendix, the few cases of imputation of birth month are not the source of the MOB artifacts discussed in this article.

Misreporting of birth dates might involve heaping at round numbers or other cognitive anchors, but even purely random error within a given time frame could introduce systematic HAZ artifacts of the type shown in Figs. 1 and 2. Random reporting of MOB within the true birth year would generate a linear gradient in HAZ from month to month within calendar years because height serves as a biological clock, advancing with true age even when age is misreported. In the standard Gregorian calendar that results in a discrete gap between December and January, or for people using other calendars, the gap would arise at their year-end, such as April (in the Nepali calendar) or September (in the Ethiopian calendar).

To see how random MOB traces out an upward gradient from month to month followed by a gap between December and January, consider a child actually born in midyear (e.g., June). The mother recalls the true birth year but not the month. If the child is erroneously recorded as born earlier in the year (e.g., January), she is actually younger than reported to be and therefore likely to be short for the reported age. If she is recorded as born later (e.g., December), she is actually older and likely to be tall for the reported age. Such errors provide a ready explanation for the anomaly shown in Fig. 1 because age is, on average, overstated for reported birth months in the beginning of the year and understated toward the end of the year.^2^ Following the same reasoning, we should find similar anomalies using weight-for-age but not with weight-for-height. Figure A3 in the online appendix demonstrates that this is indeed the case. If actual birthdays are uniformly distributed throughout the year, the only recorded birthday that would also be an unbiased estimate of the true birthday would be the midpoint of each calendar year on July 1. When dates within months are unknown, DHS protocols use the 15th of each month for this reason, but the corresponding assignment of July 1 for children without written MOB records would be infeasible.

**Fig. 4:**
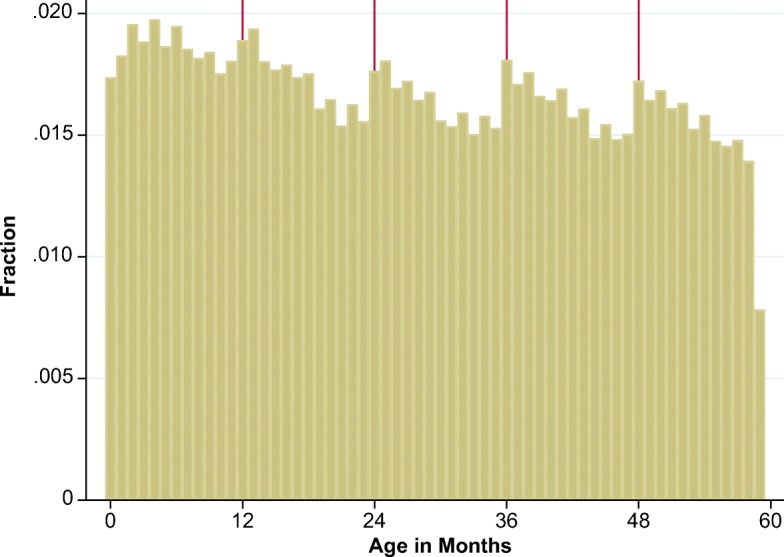
Number of births by age in months. DHS = Demographic and Health Surveys. Vertical guidelines show round ages. *Source:* DHS data for 990,231 children from 62 countries, various years.

The round-age anomaly shown in Fig. 2 is more subtle, but it could arise when enumerators start with the child’s age in completed years and then prompt for information on additional months. Consider a child who is truly 2 years and 5 months old. If the mother states the correct age in completed years but reports zero instead of five additional months, the child’s age is understated, and the child will appear tall for her age. Similarly, had the mother reported 11 additional months—that is, that her child was almost 3 years old—the child would appear short for her reported age. Hence, if the number of additional months is picked at random within the true age in completed years, it would lead to a linear gradient in HAZ sloping downward from an age in completed years until 11 additional months, similar to the calendar year gradient. But uniform random errors would imply a uniform age distribution, which is not what we see in the DHS data. Figure 4 shows classical heaping at age in completed years such as 12, 24, or 36 months, with an asymmetrical sawtooth pattern with the peak number of births reported when age in years is a round number and then declining quasi-linearly with each successive month. This suggests that the misreporting within age in completed years is not random and is not just classical heaping, but instead that ages are rounded down toward age in completed years and hence are generally understated. This implies that HAZ is upwardly biased for children with age in completed years or just above. This *asymmetric rounding* error explains why HAZ peaks at ages at round numbers and then declines to the age just below the next round age. It is consistent with interview procedures that start with an age in completed years and then add a few completed months without drawing uniformly from the entire distribution. As shown in Fig. 4, respondents are least likely to add the largest number of months, implying that the ages with the fewest reporting errors are the troughs of the age distribution, which are round ages plus 11 months.^3^

Unlike the calendar-year artifact, the round-age gap is confounded with the actual biology of linear growth in undernourished populations: because of growth faltering, we would not expect the round-age gap to be 0 even though age was correctly reported. To our knowledge, no published work has documented this phenomenon in DHS or other nutrition surveys.

## Magnitude and Significance of HAZ Anomalies

To identify the magnitude and statistical significance of anomalies that might be caused by misreported MOB, we first control only for region of the world; we then add additional tests to control for genuine socioeconomic or biological determinants of birth timing and attained heights by MOB or age in months. In each case, we present regression results graphically to visualize the data patterns. The regression results in table form are available upon request. We do not use survey weights in any of the models because the analysis focuses on subgroups by recorded MOB, for which sampling was not representative.

**Fig. 5:**
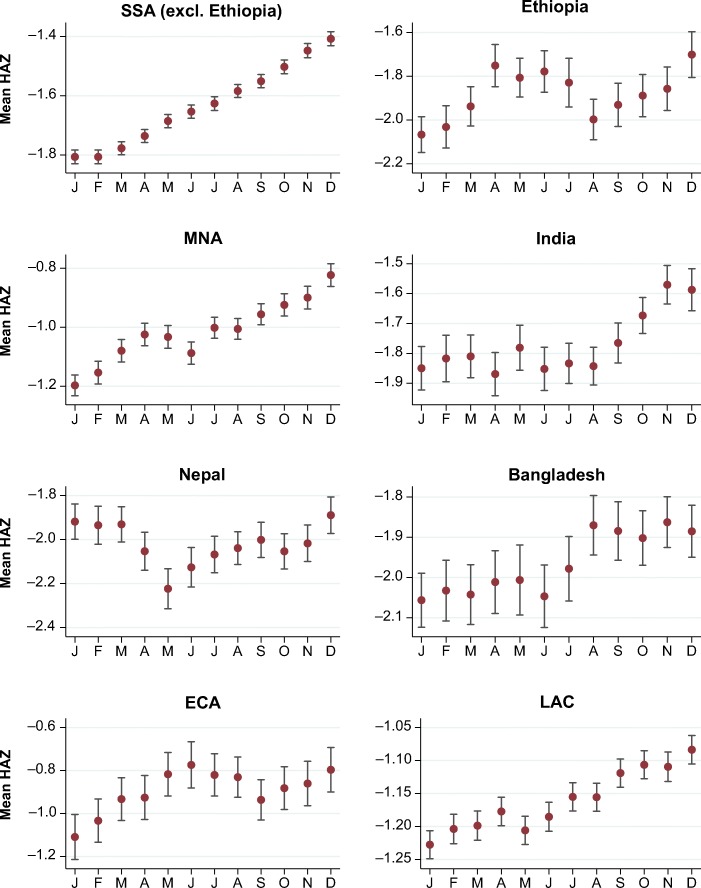
Mean height-for-age *z* scores (HAZ) by calendar month of birth (MOB) for major regions and selected countries. SSA = sub-Saharan Africa; MNA = Middle East and North Africa; ECA = eastern Europe and central Asia; LAC = Latin America and Caribbean. *Source:* DHS data for 960,012 children from 58 countries, various years.

Figure 5 reproduces the calendar-year anomaly separately for each major region of the world. This illustrates patterns previously found in regional studies, such as Dorelien (2015) for Africa or Lokshin and Radyakin (2012) for India, in the context of other regions and two countries that are of special interest because their local calendar years actually start in the middle of the Gregorian year. Nepal follows a Hindu calendar wherein each year generally begins in April, while Ethiopia follows an Orthodox calendar wherein each year begins in early September. Errors in MOB based on their calendars would not produce the same patterns we observe in other countries, and indeed the patterns in Nepal and Ethiopia are clearly more complex. In Nepal, there is a discrete break around the Hindu new year in April; but in Ethiopia, there are notable changes in the August–September period as well as the December–January period, suggesting that some respondents/enumerators in Ethiopia may use the Gregorian calendar.

The MOB gradients shown in Fig. 5 are generally steeper in poorer regions, where respondents are less likely to be literate and numerate, to have birth certificates for their children, and to celebrate birthdays for socioeconomic reasons (Stearns 2016). In sub-Saharan Africa (SSA) and the Middle East and North Africa (MNA), the December–January gap rises to a large 0.4 SD, while the gradients in the two Southeast Asian (EAP) countries (not shown) are particularly large at about 0.5 SD. But as one might expect, the gradients in eastern Europe and central Asia (ECA) and Latin America and the Caribbean (LAC) are much more modest (less than 0.2 SD in the case of LAC), although the December–January gaps are still statistically significant. These two regions have much higher maternal education and wealth levels than the remaining regions and much stronger birth registration systems. In online appendix A, we provide additional evidence on how the December–January gap is narrowed by available birth records and mother’s literacy but is unrelated to other factors.

**Fig. 6:**
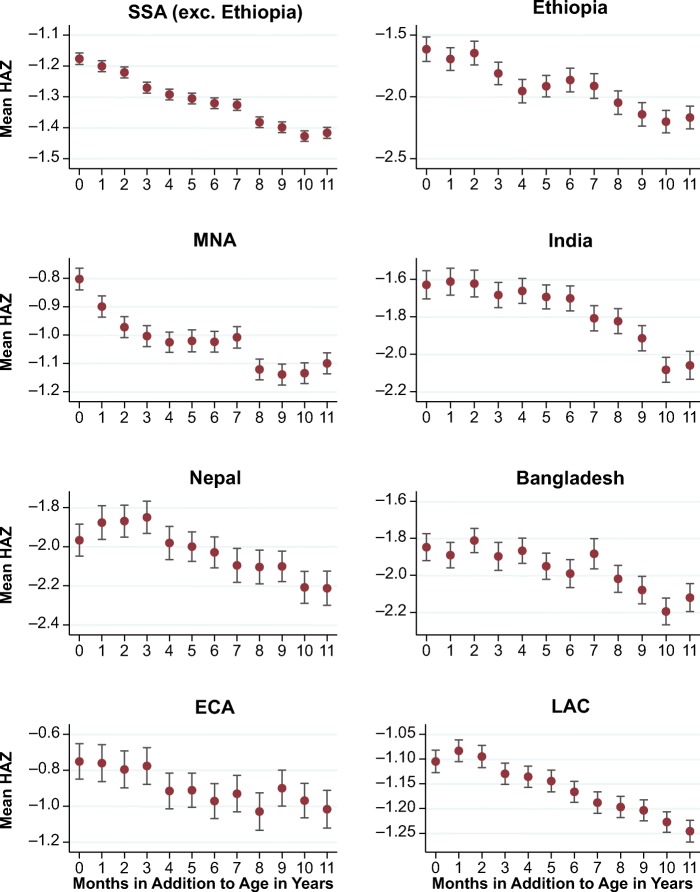
Mean height-for-age *z* scores (HAZ) by additional months for major regions and selected countries. SSA = sub-Saharan Africa; MNA = Middle East and North Africa; ECA = eastern Europe and central Asia; LAC = Latin America and Caribbean. Corresponding graphs where controls are included can be found in online appendix Fig. A14. *Source:* DHS data for 960,012 children from 58 countries, various years.

Figure 6 shows the round-age anomaly by region and country, for ease of comparison with the Fig. 5 (regression results are available upon request). A notable difference is that Ethiopia and Nepal, the two countries for which national calendar systems affected the gradient in Fig. 5, are not particularly distinctive. That observation is consistent with errors due to asymmetric rounding being substantially independent from random MOB errors because the former emerges relative to round ages rather than calendar months.^4^

Before turning to multivariate regressions, we relate our results to two indicators of measurement error used in previous work. The first step is to compare our December–January gap with the SD of HAZ. The SD of HAZ could reflect genuine dispersion related to health inequality but is widely used as an indicator of survey errors in both height and age (Assaf et al. 2015; Mei and Grummer-Strawn 2007). Meanwhile the December–January gap is confined to capturing errors in MOB, but it remains unconfounded by health inequality.

**Fig. 7:**
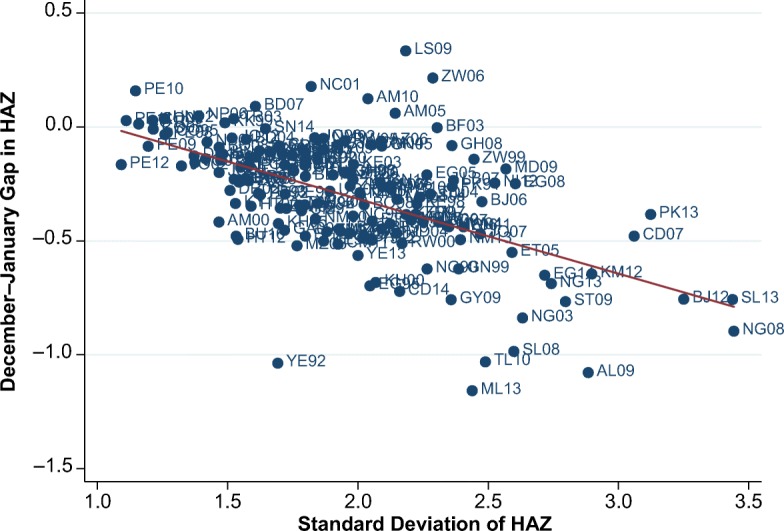
Magnitude of December–January gap by standard deviation of height-for-age *z* scores (HAZ) in 163 surveys. *Source:* DHS data for 990,231 children from a total of 163 surveys across 62 countries, various years.

Figure 7 reveals a strong association between the magnitude of the December–January gap and the SD of HAZ, with a correlation of –.56 that is significant at the 1 % level. All countries and years are labeled to identify outliers. Peru has previously been identified as a country implementing high-quality DHS (Assaf et al. 2015), and it stands out on the far-left side of the distribution, with a low SD and a small December–January gap, especially in the 2012 survey. At the opposite end, Nigeria in 2008 and Sierra Leone in 2013 have high levels of both kinds of error. Outliers above the regression line have relatively low or even reversed December–January gaps, while those below the line have large December–January gaps. This finding suggests that misreporting birth months contributes heavily to overall measurement error, as in Yemen 1992, Timor-Leste 2010, and Mali 2013.

Another commonly used indicator for measurement error is age heaping (Assaf et al. 2015), which in our data occurs primarily around round-numbered ages, as shown in Fig. 4. The measurement error that causes heaping in ages is generally considered to be symmetric around the cognitive anchor; as a result, HAZ measures are unbiased but noisier. When we couple the age distribution with mean HAZ by age in months, we can conclude that HAZ is upwardly biased for children with round ages or just above round ages because the age is underreported. However, the round-age gap cannot be used as a clear metric for this measurement error because it is confounded by true growth faltering. Using regression analysis, we demonstrate that the two types of errors are independent: December–January gap does not capture errors caused by asymmetric rounding, and hence, as a metric for measurement error, it provides a lower bound for the degree of measurement error in age.

To isolate the anomalies in HAZ after controlling for a wider range of possible influences, we provide a set of estimates using the following regression specification:

1 $$ {H}_i=\sum \limits_{m=1}^{11}{\upbeta}_m{\mathbf{MOB}}_{m,i}+\sum \limits_{d=1}^{11}{\upgamma}_d{\mathbf{months}}_{d,i}+\updelta {D}_i+\uptheta {\mathbf{X}}_i+{\boldsymbol{\upmu}}_s+{\upvarepsilon}_i, $$

where *H*_*i*_ is HAZ for child *i*; **MOB**_*m*, *i*_ are the 11 month-of-birth dummy variables, with December as reference category; and **months**_*d*, *i*_ are the 11 dummy variables for the age in months in addition to a round age, with 0 (that is, a round age) as the reference category (e.g., a 27-month-old girl is 3 months in addition to her round age of 2 years). **D**_*i*_ is child demographics (child gender, age, and age squared); **X**_*i*_ is a series of parental/household control variables (household assets, parental education, total number of children, total number of adults, toilet availability, water source, and a rural dummy variable); **μ**_*s*_ refers to survey dummy variables for each country and survey wave; and ε_*i*_ is the error term that we cluster at enumeration areas.

**Fig. 8:**
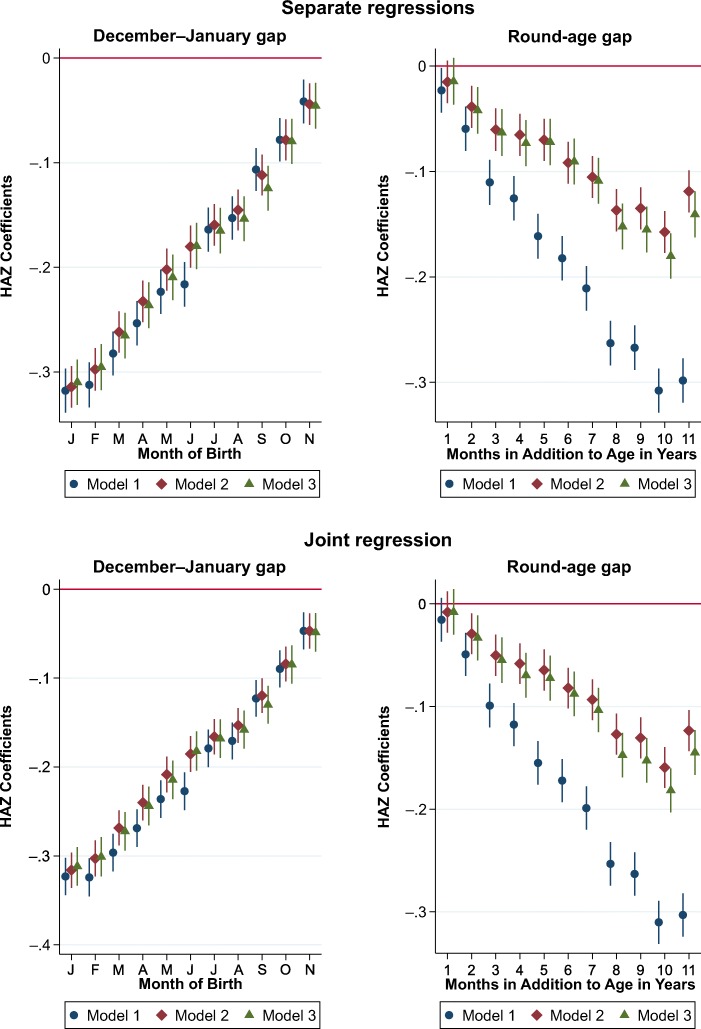
Separate and joint tests for month-of-birth (MOB) and round-age biases in height-for-age *z* scores (HAZ) scores with alternative sets of control variables. The graphs present coefficients to MOB dummy variables from three regressions: raw means (Model 1), controlling for child demographics and survey fixed effects (Model 2), and controlling for parental and household characteristics (Model 3). *Source:* DHS data for 990,231 children from 62 countries, various years.

Results of our multivariate econometric tests are presented using the same type of visualization as the bivariate relationships, first in terms of calendar months (and hence the December–January gap) and then in terms of months in addition to a round age (and hence the round-age gap). Regressions use the specification from Eq. (1), varying the statistical controls included in the model. We estimate separate models for each kind of artifact (excluding either **MOB**_*m*, *i*_ or **months**_*d*, *i*_) and then a combined model that includes both calendar months and months in addition to age in years. For each of these two specifications, we initially add no controls (Model 1); then control for child demographics and survey fixed effects, **D**_*i*_ and **μ**_*s*_ (Model 2); and then also control for all available socioeconomic factors, **X**_*i*_ (Model 3). We summarize the results of these regression estimates in Fig. 8. Complete regression results are available upon request.

The main conclusions from results shown in Fig. 8 are threefold. First, the calendar MOB bias is entirely robust to confounding factors: a December–January gap of approximately 0.3 SD persists even after we control for age and socioeconomic factors. Second, the round-age gap does appear to include both an artifactual component and an actual decline: the magnitude of the gap falls from 0.3 SD to 0.13 SD after we control for child age. Third, these two biases appear to exist independently: specifying both sets of dummy variables in the model has no effect on the coefficients of either set.^5^

## Simulation Results

To demonstrate how misreporting MOB can explain the observed calendar-year anomaly in HAZ, we introduce random noise in a known population and quantify how much error would be needed to explain the December–January gap in HAZ observed in actual DHS data. This simulation model begins by replicating the WHO reference population of height-for-age, adjusts heights to match the mean and standard deviation observed in the global DHS sample, and then introduces random birth months to a varying fraction of those children to replicate the December–January anomaly. Because the DHS data span all regions of the world with very diverse agroecologies and seasonal weather patterns, we expect that the true December–January gap would be 0 (or at least close to 0) in absence of measurement error. We abstain from reproducing the round-age gap because it is confounded with age; hence, we would not expect the gap to be 0 even when age is correctly reported. The simulation of the December–January gap is not affected by omitting the round-age gap because the two anomalies are independent, as demonstrated in Fig. 8. The fact that we are simulating only part of the error that we have detected in the data suggests that we are providing a lower bound for the misreporting apparent in the data.

Our simulation approach reveals the fraction of children for whom a purely random birthday generates the observed anomaly. In practice, we acknowledge that many parents may make nonrandom estimates of their child’s MOB (e.g., they may know it was in the first half of the year), but the stylized dichotomy of random/true MOBs can at least demonstrate how random noise in birthdays creates a measurable trace in HAZ data. By comparing the reference distribution calibrated to DHS data with and without error in reported birth dates, we can also estimate the extent to which those errors affect stunting rates.

Online appendix B contains a detailed protocol for these simulations, so here we provide only the basic details and intuition of the simulation approach. The benchmark heights used in the simulations are drawn from normal distributions for a female population at each age in days. The mean and the standard deviation of the distribution at each age are derived from the WHO child growth standards for girls (WHO MGRSG 2006), transformed to fit the observed level and variation of actual heights in the DHS. To replicate smoothed growth velocities observed in the DHS, our benchmark population has its growth velocity slowed relative to WHO standards by 7 % less growth each day from 0 to 6 months of age, then 21 % less growth each day from 6 to 24 months of age, and finally 10 % less growth from 24 months onward.^6^ To adjust the SDs of height, we add 2 to the WHO SDs to approximate a realistic spread in the DHS HAZ distribution. We motivate this adjustment as a combination of overall measurement error (i.e., not related to the aforementioned artifacts) and increased dispersion due to variation in nutritional status of the children in the sample. We furthermore multiply the WHO SDs by 0.85 to account for the fact that the SDs in the DHS data increase less with age than the WHO SDs (a potential explanation for which could be that errors in height measurements decrease with age). We choose this specification to fit the simulated stunting rates to those in the DHS data and because the measurement errors that we simulate also increase the SD of height.

The first step in replicating random MOB errors involves drawing a random birth month and birth day for each child in the simulated data. We then calculate the age with measurement error based on the random month and day along with a true birth year, and we use this random MOB age to calculate an HAZ with error. Note that we include only children with an erroneous age below 60 months given that the sample selection in the DHS surveys is based on the reported age, which implies that some children who are truly older than 60 months are included in the sample while some children truly below 60 months of age are excluded from the sample. We then map the true simulated HAZ and the HAZ with error by the reported birth month (either true or random).

**Fig. 9:**
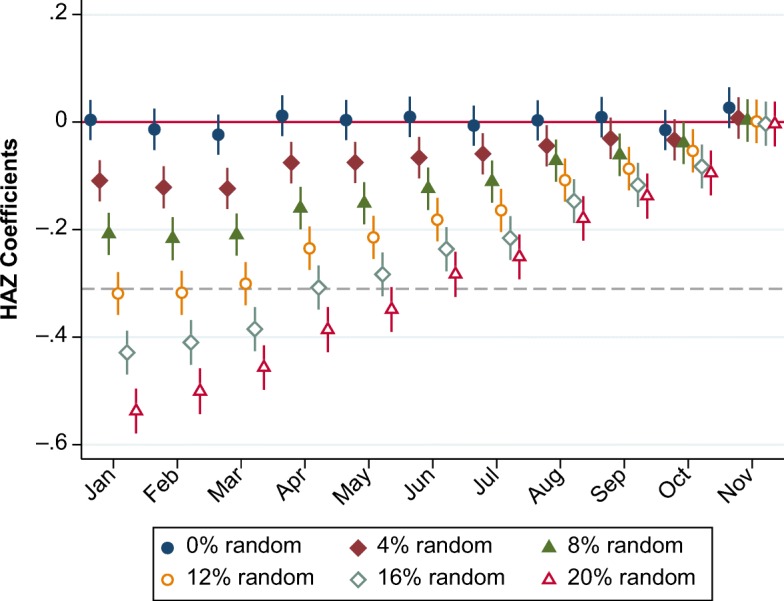
Simulated height-for-age *z* scores (HAZ) by share of children with random month of birth. The dashed line represents December–January gap in DHS data. *Source:* Simulated data.

The magnitude of the artifact created by varying the share of children affected by random MOB is shown in Fig. 9. This reveals how assigning random birth months yields almost exactly the same pattern observed in the actual DHS data, with a December–January gap in mean HAZ whose size reflects the share of children with a randomly assigned MOB. We can vary the fraction reporting a random birth month to find the share that fits the actual December–January gap of –0.32 SD found in the DHS data.

Our calibration procedure is illustrated in Fig. 9 and detailed in Table B1 in the online appendix. The observed worldwide gap of exactly –0.32 SD can be replicated by reassigning 11 % of the sample to a random MOB. After doing so, we find an overall stunting rate of 35.7 % compared with 35.2 % in the true data and a severe stunting rate of 15.2 % compared with 14.5 % in the true data, suggesting that the bias in stunting rates due to MOB misreporting is rather small in the overall DHS data. This random MOB artifact will generally bias stunting estimates upward in poorer and less-educated populations, and it could slightly inflate the stunting differences between different socioeconomic groups (e.g., poor and nonpoor, educated and non-educated). However, this upward bias in stunting will also be partially offset by the bias due to the second anomaly, which results in children being reported as younger than they actually are, on average, and thereby appearing taller for their age than they actually are. Overall, we conclude that significant biases in stunting estimation are likely to be confined to some of the lowest-quality surveys in the DHS. Table B2 in the online appendix lists each DHS survey used and the size of its December–January gap in HAZ. Of the 163 surveys used, 59 surveys are estimated to have 10 % to 20 % of sampled children reporting a random MOB, 15 surveys are estimated to have 20 % to 30 % reporting a random MOB, and 6 surveys are reported to have 30 % to 41 % of their sample reporting a random MOB. Notably, in four rounds of surveys in Nigeria—the most populous country in SSA—we find that the shares reporting random MOB exceed 20 %, which also leads to larger biases in stunting estimates in Nigeria (up to 1.3 percentage points and 2.0 percentage points for overall and severe stunting, respectively).

**Fig. 10:**
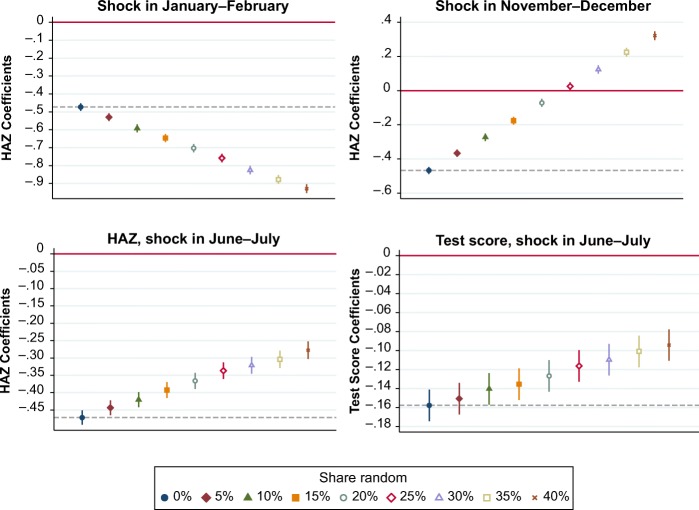
Bias in estimated impacts on attained height-for-age *z* scores (HAZ) or a test score of a simulated known shock, by time of year. Data shown are the estimated impact on measured HAZ and test score of “exposure” to an *in utero* shock that occurs at the start, middle, or end of a calendar year. The true effect size is –0.48 for HAZ and –0.16 for the test score. Increasing the fraction of children whose birth date is misreported reveals asymmetry in the bias that arises from introducing purely random errors in month of birth. For shocks that occur early in the year (shown here as January and February), misreporting leads to younger and hence shorter children being erroneously recorded as exposed to the shock, thereby overstating its negative effect on HAZ. For shocks that actually occur later in the calendar year (e.g., November and December), misreporting leads to older and hence taller children being erroneously recorded as exposed to the shock, thereby offsetting its true negative effect on HAZ and potentially reversing its sign. For shocks that occur in around July 1 (here, in the months of June and July), children misreported to have been exposed are equally likely to be older or younger than their recorded age, so misreporting introduces only attenuation bias toward an effect size of 0. Similar attenuation bias is found when the outcome is a test score, regardless of the timing of the shock. *Source:* Authors’ calculations, from simulated DHS data and simulated test score data.

In Fig. 10, we report how our simulation approach can be used to identify the bias introduced by age misreporting when estimating the effect of an early-life shock on attained height and on a test score. We employ the simulated height distribution as described earlier and also simulate a normal test score distribution with a mean of 50 and a SD of 10. In these synthetic case studies, we introduce a negative shock *in utero* to which only some of the children in our simulated population are exposed. The known shock we impose is designed to mimic a monsoon-type event that lasts for two months of the year and is harmful only to infants who are born at that time. Exposed children have a 3 % lower birth length than unexposed children, which we deduct before calculating HAZ. Similarly, test scores are 3 % lower, which we deduct before standardizing the test score by subtracting the mean and dividing by the SD. Exposure depends on the child’s true MOB, with the frequency of misreporting and hence magnitude of bias shown along the horizontal axis. The effect size is shown along the vertical axis. In this population, our synthetic shock has a true average treatment effect of –0.48 HAZ points or –0.16 standardized test score points, which is accurately estimated when the percentage of misreporting is 0. These regression coefficients are represented as blue dots, and their level is indicated by dashed horizontal lines in the graphs.

The figure contrasts three possible directions of bias that could be introduced in HAZ coefficients, depending on when the shock actually occurs relative to the misreporting of birth months. Recall that misreporting introduces a downward bias to HAZ for children recorded as born early in the calendar year and an upward bias to HAZ for those recorded as born late in the year. On average, if births are uniformly distributed through the calendar year, only children who are recorded as born on July 1 would have an unbiased level of HAZ. Any bias is added to the true shock, so the direction of bias depends on whether the shock actually occurs early or late in the calendar year. For shocks that actually occur midyear, the only bias is attenuation toward 0 because of misclassification into exposure to the shock. For all outcomes for which age is not an integral part of the variable, regression coefficients will be attenuated toward 0 regardless of the timing of the shock.

Each point estimate shown in the graphs in Fig. 10 results from regressing HAZ or a test score on “exposure” to a shock, where classification into exposure depends on the reported MOB. The upper-left graph of this figure illustrates how this negative effect is systematically overstated by age misreporting when the true shock occurs early in the calendar year, in January and February. In that case, some of the children recorded as “exposed” are actually younger and hence shorter than their reference population, so their downward misreporting bias is added to the true negative effect of the actual shock. The upper-right graph of Fig. 10 shows the opposite case, when the true shock occurs in November and December, so some of the “exposed” children are actually older and hence taller, thereby offsetting the estimated shock effect. In this specific case, when the rate of misreporting exceeds approximately 24 % of all children, the positive bias is larger than the negative shock, thus reversing its direction of estimated effect.

The lower-left graph in Fig. 10 traces out the case of pure attenuation bias that is introduced when the true shock is uniformly distributed around the midpoint of each calendar year, in June and July. Similarly, the lower-right graph of the figure illustrates the attenuation bias when the outcome variable is a simulated test score, demonstrating that misreporting of MOB is a problem not only for research on HAZ but also more broadly for all research designs that rely on MOB to classify individuals into exposure to a shock in early childhood.

## Discussion

In this study, we examine the consequences of mismeasured MOB for child HAZ scores. Using all suitable DHS data from 1990 to 2014, we find two puzzling anomalies that can readily be explained by two related kinds of measurement error, especially in countries and regions with low levels of maternal education, low socioeconomic status, and incomplete birth registration. First, the *calendar-year anomaly* is a gradient in height-for-age by calendar MOB and an implausibly large gap between children whose reported birth dates fall at the end of one calendar year and those with birth dates at the start of the next. Second, the *round-age anomaly* is a gradient in height-for-age by number of months after an age in completed years and an implausibly large gap between children whose reported age is a completed year plus 11 months and those whose age is the next completed year. The first can be explained as an artifact arising from random or quasi-random recording in calendar MOB within the true birth year, while the second appears to stem from a tendency for enumerators/respondents to round down age toward age in completed years. The two biases appear to operate independently.

Our central finding is that the calendar-year anomaly, measured as the December–January gap in HAZ by MOB, can be used to measure the fraction of children whose birth month is misreported. Attained height is informative because linear growth serves as a biological clock, advancing with true age even when age is misreported. What are the implications of these results for nutritional research? We identify four main areas of concern.

First, errors in MOB could cause a bias in stunting rates. Errors in calendar MOB do not affect median HAZ but do increase the spread of the HAZ distribution, leading to fatter tails and more children recorded to the left of the –2 SD cutoff. In contrast, errors in age in months relative to round ages in completed years lead to underreporting of age, on average, and hence overestimation of HAZ scores. Although these two biases work in opposite directions, we caution against ignoring these errors because particular surveys may be more predisposed to one type of error than the other and because the prevalence of these errors can be very high in particular surveys (e.g., Nigeria) or for particular subgroups.

Second, errors in MOB are potentially a good indicator of the quality of anthropometric and demographic data and perhaps of overall survey quality. The most commonly used measure of error in HAZ measurement, the SD of HAZ, has the inherent limitation of reflecting genuine dispersion (inequality in nutrition) as well as artificial dispersion (errors in measurement). In contrast, the HAZ-MOB gradient and the December–January gap in HAZ do not suffer from this limitation, and they are readily implemented tests that can be used to identify the extent of bias and to gauge the effectiveness of attempts to improve data quality. Coupled with the results reported in online appendix B, the December–January gap can be used to estimate the proportion of any given sample of children that may have been assigned a random birth month. Moreover, although our study focuses only on DHS surveys, these measurement error problems may be even more pronounced in multipurpose surveys in which the challenges of measuring children’s age and height accurately are paid even less attention. Indeed, we find similar errors in the Living Standards Measurement Study surveys implemented by the World Bank and in Multiple Indicator Cluster Surveys implemented by UNICEF (results are available upon request). In these and future surveys, our results provide an easily implementable approach to quickly checking for measurement error in MOB.

Third, errors in MOB could affect studies that use birth timing to estimate the impacts of known events on child height and stunting. Studies from developed countries with highly accurate birth registration exploit timing of exposure to gauge the long-term impacts of shocks very effectively (Currie and Rossin-Slater 2013; Deschenes et al. 2009; Messias et al. 2006). Clearly, one would expect developing-country populations to be far more vulnerable to shocks because poor populations are unable to protect themselves and because these populations are generally exposed to more severe shocks more frequently (Chambers 1982). Many studies on developing countries have attempted to gauge the nutritional impacts of agroclimatic shocks (Brainerd and Menon 2014; Mulmi et al. 2016; Tiwari et al. 2017), economic and political shocks (Akresh et al. 2011; Baird et al. 2011; Bhalotra 2010; Block et al. 2004), or fasting among pregnant women (Almond and Mazumder 2011; Majid 2015). It is likely that these studies suffer from attenuation bias because nontrivial proportions of children will be classified as exposed to a shock when in fact they were not exposed (or vice versa).

Fourth, errors in MOB have serious implications for studies of the impact of birth seasons on long-run child growth. Studies of rural Gambian communities, which circumvented age-misreporting problems by focusing on indicators of birth outcomes, do indeed suggest the existence of strong seasonal determinants of children being born prematurely or being short for gestational age and of poor maternal weight and anemia during pregnancy (Prentice et al. 1981; Rayco-Solon et al. 2005), and a handful of similar but much earlier biological studies were conducted in other developing countries (for a review, see Rayco-Solon et al. 2005). However, recent studies examining the longer-run HAZ effects of birth seasons are inherently flawed by measurement error in MOB because these errors in age implicitly appear on both the left side of the equation in HAZ and on the right side as a reported MOB (as in Dorelien 2015; Lokshin and Radyakin 2012). These studies have erroneously inferred that births in earlier months in the calendar lead to worse HAZ outcomes and therefore warrant season-specific interventions, such as safety nets or additional public health interventions. Although there may indeed be true seasonality in HAZ outcomes, the errors in DHS and similar surveys arguably create an insurmountable confounding problem.

Finally, our results identify age measurement as a difficult challenge for surveys implemented in underdeveloped settings and suggests two areas for further research: (1) deriving correction factors based on misclassification of MOB in order to adjust for attenuation bias and directional bias in future studies employing error-prone data; and (2) improving measurement of children’s age in these settings through further experimentation in the field. To improve measurement, some avenues for exploration include the adoption of more sophisticated event calendars, the questioning of both mothers and fathers, and more intensive training of enumerators to avoid the serious consequences of age misreporting documented in this article.

## Electronic supplementary material

ESM 1 (PDF 1496 kb)

## Footnotes

1 For more than 97 regression coefficients for each of three models reported in Dorelien (2015: appendix), the median coefficient is 3.1, and the mean is 2.87.

2 We argue that the error in MOB is uniform over the year given that we do not see a consistent tendency to report months earlier or later in the year; see online appendix Fig. A2.

3 Consistent with this, online appendix Fig. A4 shows that the child age is significantly correlated with mother’s education: mothers who report a round age for a child are less educated than those who report a round age plus 11 months.

4 Online appendix Fig. A14 also shows these results after controlling for additional covariates.

5 In Fig. A15 of the online appendix, we repeat this exercise for whether a child can be classified as stunted (HAZ < −2). The artifacts are again large: the December–January gap is just over 5 percentage points, irrespective of the model, and children reported as being 11 months older than a round age are 2 to 3 percentage points more likely to be classified as stunted compared with children with a round age.

6 This benchmark growth trajectory corresponds to the means in the DHS data as shown in Fig. B1 (online appendix). In those data, a negative bump in simulated heights just after 730 days of age (2 years) is linked to the fact that children after that age are measured standing rather than recumbent. According to the WHO growth standards, standing height is 0.7 cm shorter than recumbent length. Figure B2 illustrates that the simulated SDs fit the slope of the SDs in the DHS data, although they are in general 1.5 times lower. The resulting simulated true HAZ by age is shown in Fig. B3, which is smoother than the DHS HAZ by age.
