# Supplementary material for: Misreporting Month of Birth: Diagnosis and Implications for Research on Nutrition and Early Childhood in Developing Countries
Source: Demography. 2019 Jan 28;56(2):707–28. doi: 10.1007/s13524-018-0753-9 (PMC6449488; doi:10.1007/s13524-018-0753-9)
Supplement: Supplementary file 1 — (PDF 1496 kb) [file 13524_2018_753_MOESM1_ESM.pdf]

Online Appendix

This document includes supplementary material for

Misreporting Month of Birth: Diagnosis and Implications for Research on Nutrition and Early Childhood in Developing Countries

by

Anna Folke Larsen, Derek Headey and William A. Masters

Published in Demography

A: Supplementary results

Table A.1 Sample sizes for anthropometric data by region for 62 countries

Region	Countries	Child observations	Frequency
East Asia and Pacific	2	19,447	2.0%
Europe and Central Asia	7	18,653	1.9%
Latin America and Caribbean	10	222,255	22.4%
Middle East and North Africa	5	145,081	14.7%
South Asia	5	98,260	9.9%
Africa south of the Sahara	33	486,535	49.1%
Total	62	990,231	100.0%

Source: ICF International (2015).

Figure A.1 HAZ by MOB for children with and without imputed birth month

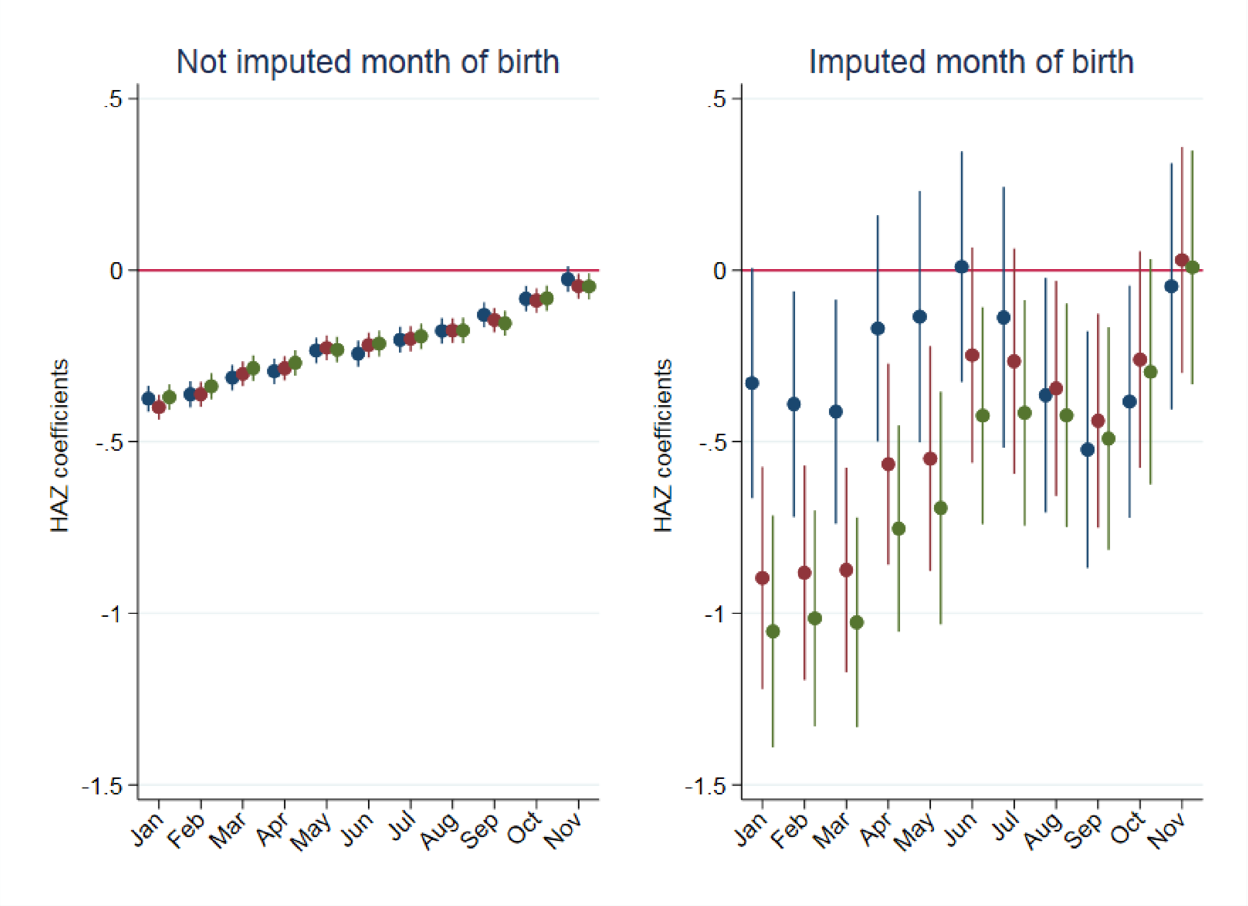

Source: DHS data from 396,299 children in 17 countries in Africa south of the Sahara and Egypt and India.

Note: HAZ = height-for-age z-scores; MOB = month of birth. Two percent of children have imputed month of birth. The graphs present coefficients to MOB dummies from three regressions: raw means (in blue); controlling for child demographics and survey fixed effects (red); and also controlling for parental and household characteristics (green). Regression results are available upon request.

Figure A.2 Number of births by MOB

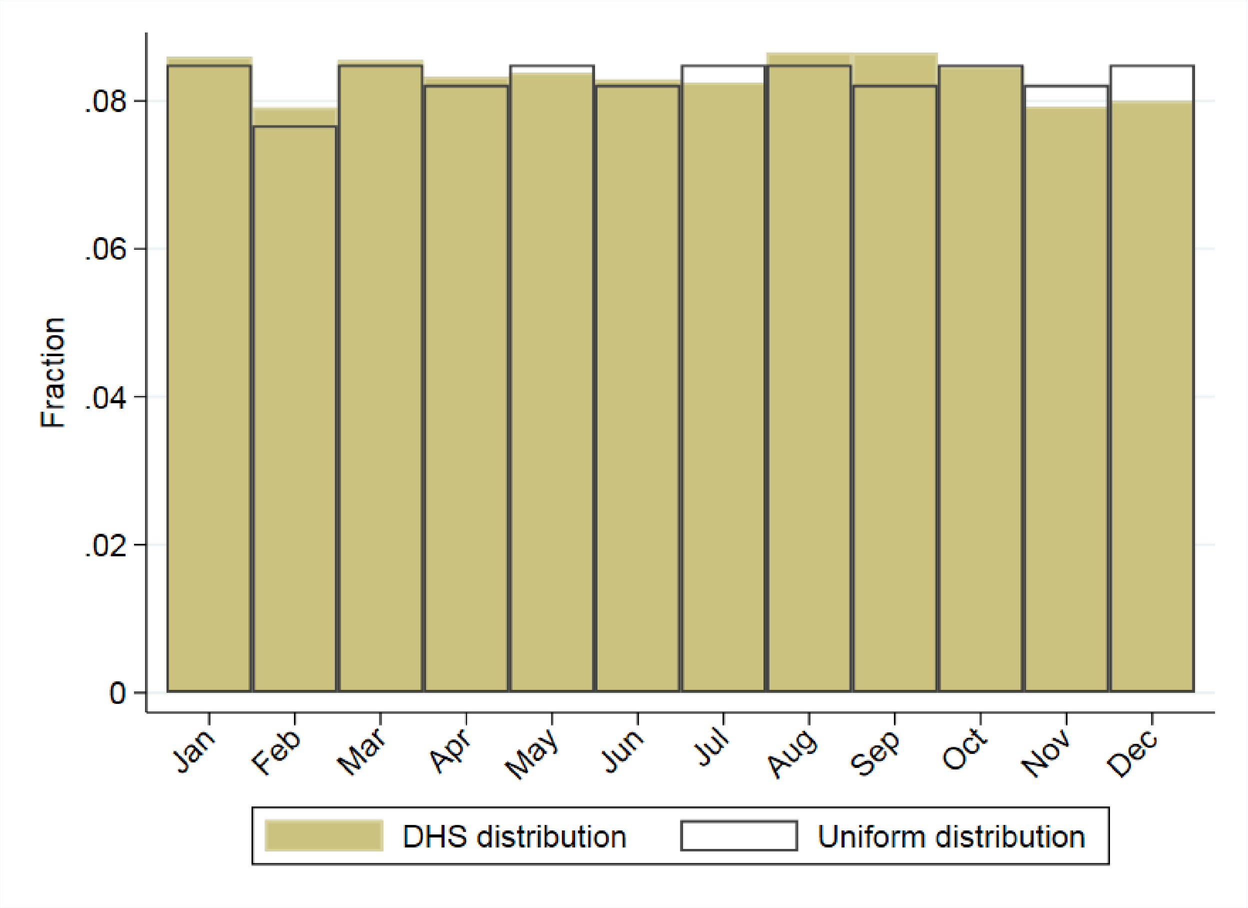

Source: DHS data from 990,231 children in 62 countries, various years.

Note: DHS = Demographic Health Surveys. The uniform distribution illustrates the distribution of birth months when birthdays are reported with equal probability for all days of the year.

Figure A.3 Weight-for-Age Z-score (WAZ) and Weight-for-Height Z-score (WHZ) by MOB

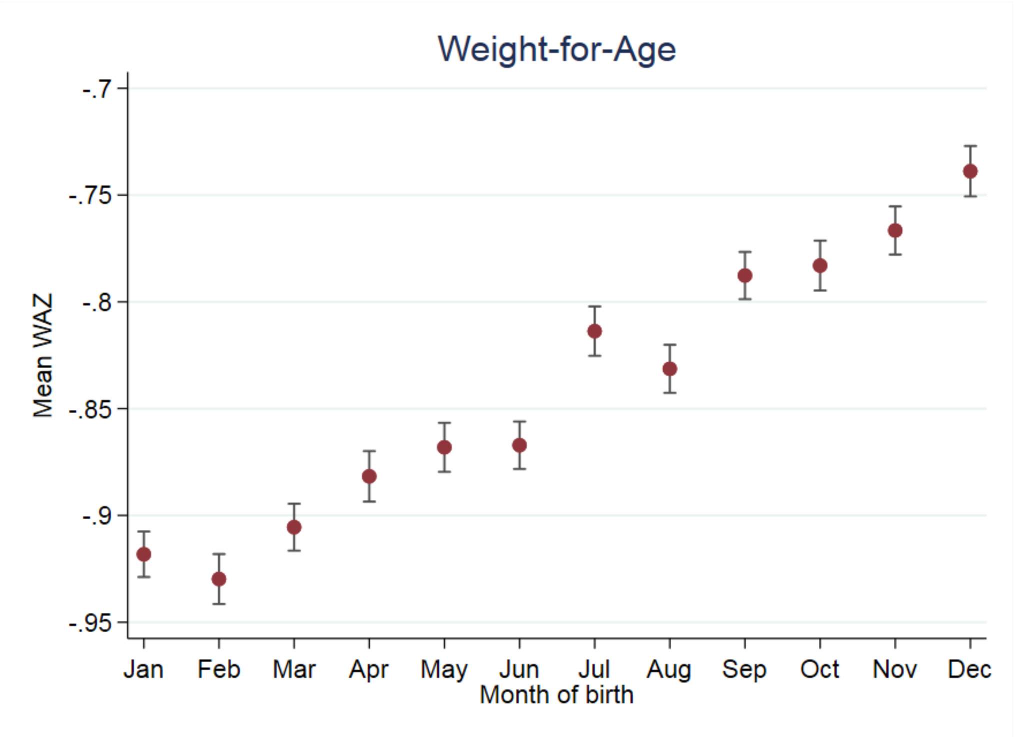

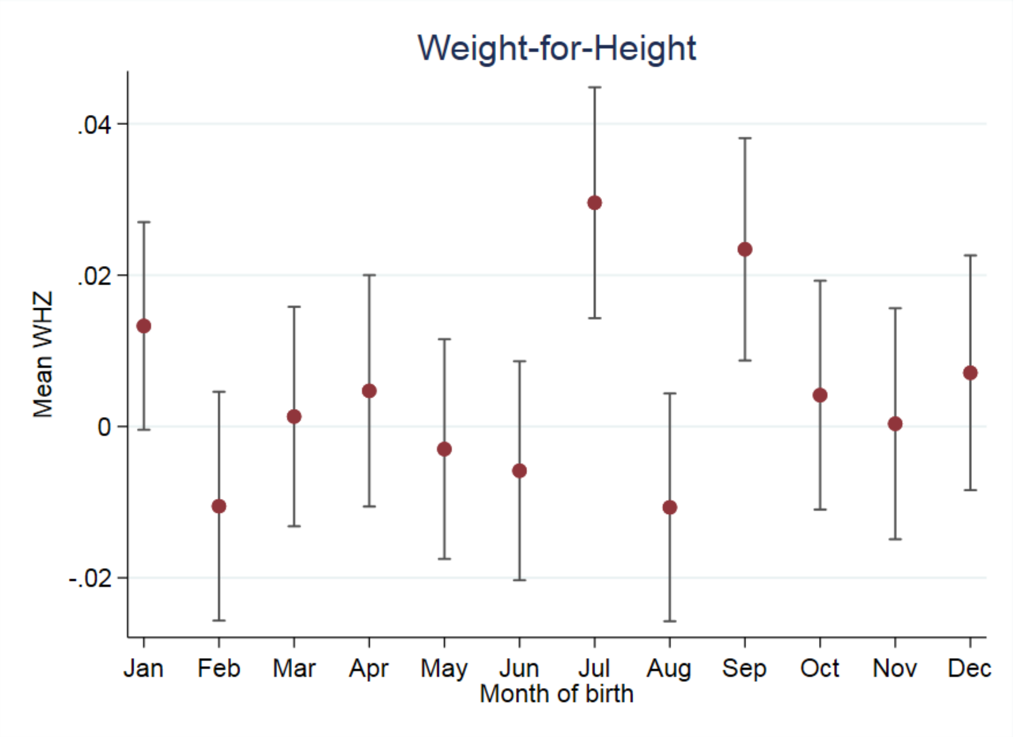

Source: DHS data from 982,666 (WAZ) and 976,278 (WHZ) children in 62 countries, various years.

Figure A.4 Mother's education in years by months in addition to age in years

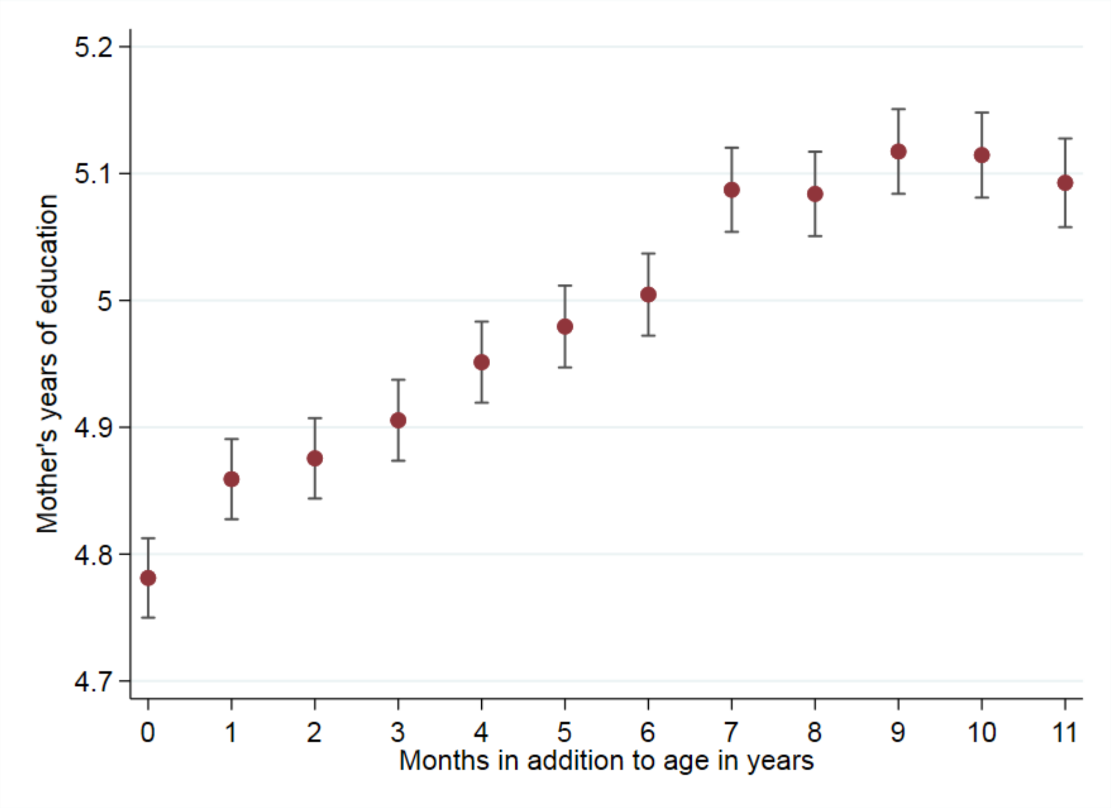

Source: DHS data from 975,534 children in 62 countries, various years.

Figure A.5 HAZ–MOB gradients for major regions and selected countries with controls

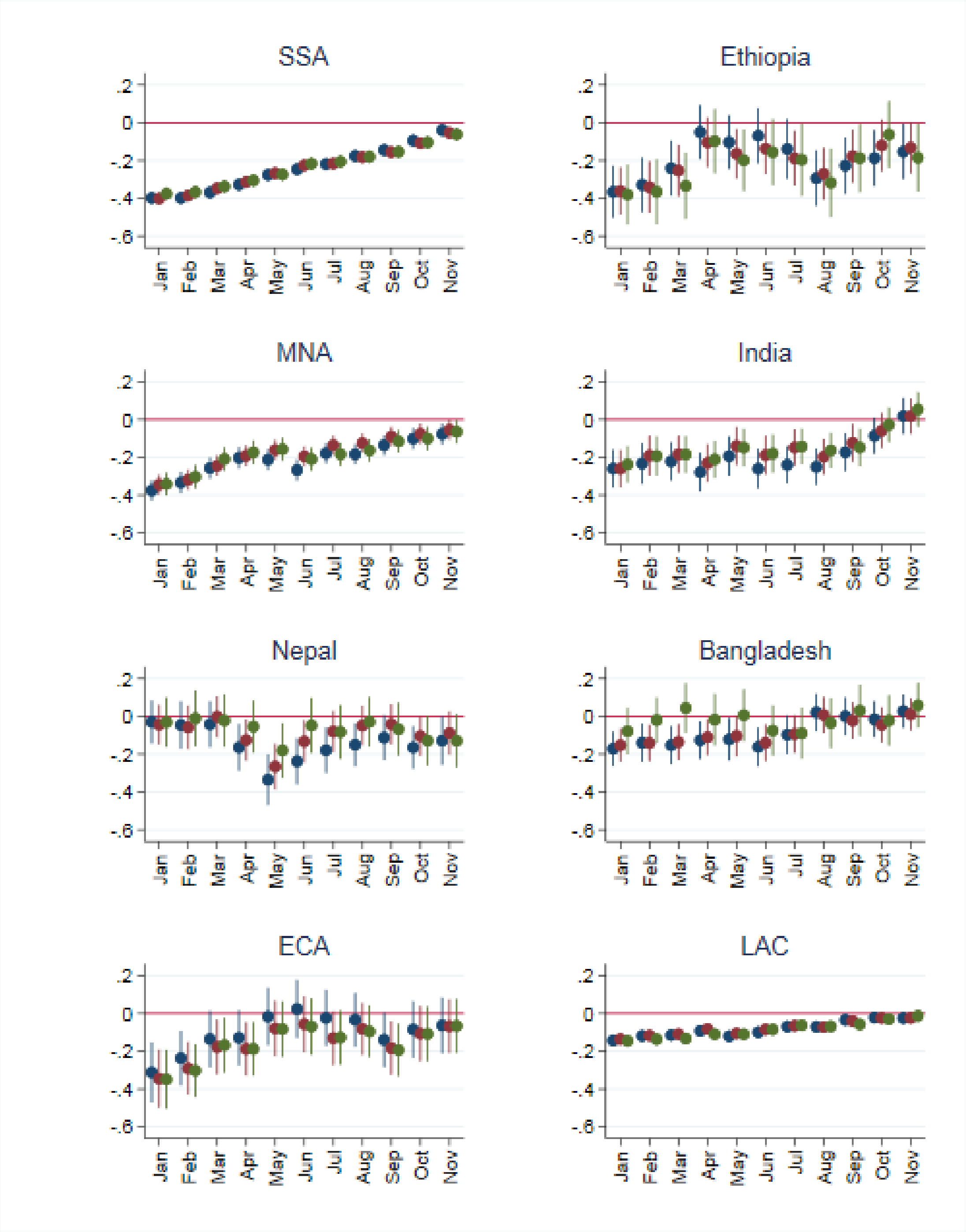

Source: DHS data for 960,012 children from 58 countries, various years.

Note: HAZ = height-for-age z-scores; SSA = Africa south of the Sahara; MNA = Middle East and North Africa; MOB = month of birth; ECA = Eastern Europe and Central Asia; LAC = Latin America and Caribbean. The graphs present coefficients to MOB dummies from three regressions: raw means (in blue); controlling for child demographics and survey fixed effects (red); and also controlling for parental and household characteristics (green). Regression results are available upon request.

Figure A.6 HAZ by MOB depending on the mother's education

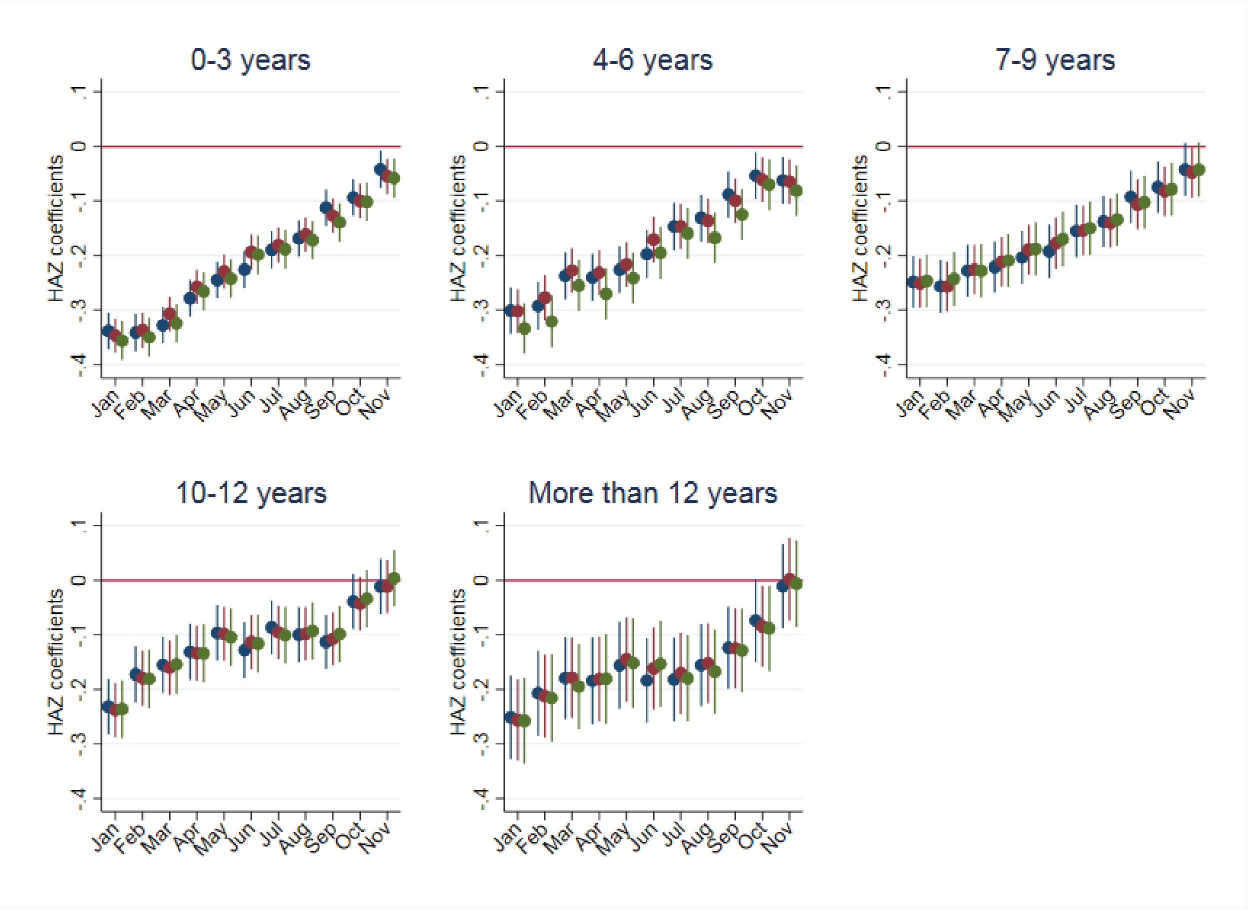

Source: DHS data from 975,534 children in 62 countries.

Note: HAZ = height-for-age z-scores; MOB = month of birth. Forty-four percent of children have mothers with 0–3 years of schooling; 19 percent have mothers with 4–6 years of schooling; 16 percent have mothers with 7–9 years of schooling; 14 percent have mothers with 10–12 years of schooling, and 6 percent have mothers with 13 or more years of schooling. The graphs present coefficients to MOB dummies from three regressions: raw means (in blue); controlling for child demographics and survey fixed effects (red); and also controlling for parental and household characteristics (green). Regression results are available upon request.

Figure A.7 HAZ by MOB depending on the mother's literacy

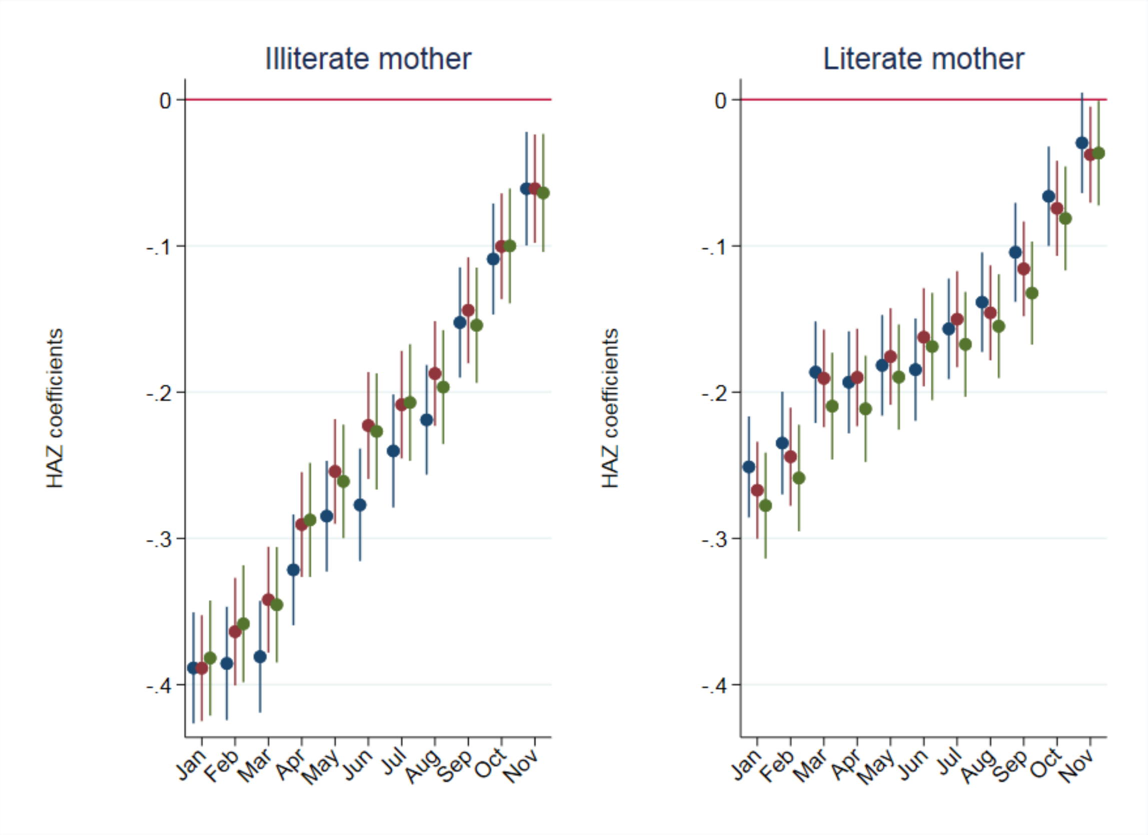

Source: DHS data from 395,347 children in 50 countries, various years.

Note: HAZ = height-for-age z-scores; MOB = month of birth. Fifty-three percent of the children have illiterate mothers; 47 percent have literate mothers. The graphs present coefficients to MOB dummies from three regressions: raw means (in blue); controlling for child demographics and survey fixed effects (red); and also controlling for parental and household characteristics (green). Regression results are available upon request.

Figure A.8 HAZ by MOB depending on whether the mother has shown the child's birth certificate

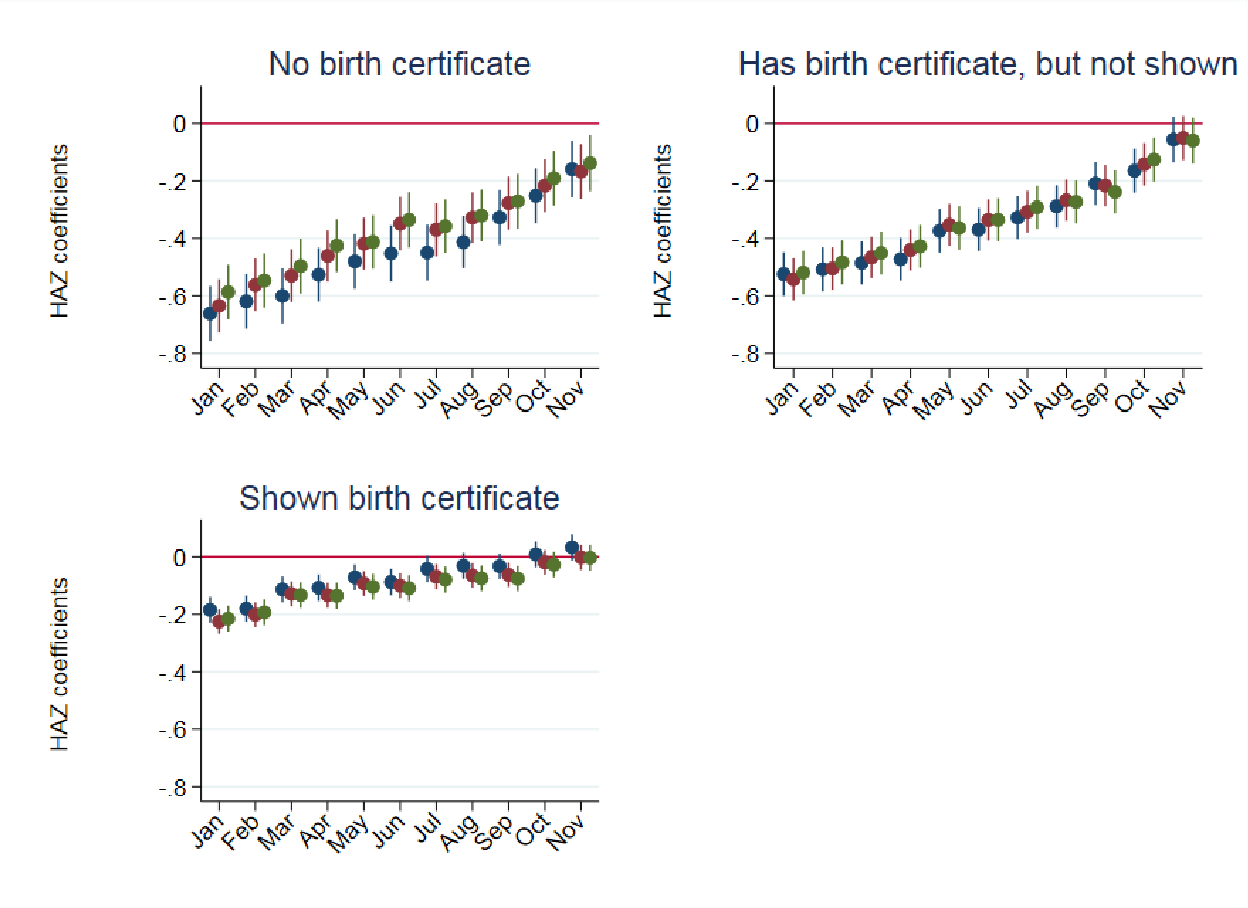

Source: DHS data from 396,299 children from 17 countries in Africa south of the Sahara and Egypt and India.

Note: HAZ = height-for-age z-scores; MOB = month of birth. Twenty-one percent of children have no birth certificate; 26 percent have a birth certificate but it is not shown to the enumerator; and 53 percent show the birth certificate. The graphs present coefficients to MOB dummies from three regressions: raw means (in blue); controlling for child demographics and survey fixed effects (red); and also controlling for parental and household characteristics (green). Regression results are available upon request.

Figure A.9 HAZ by MOB by age group

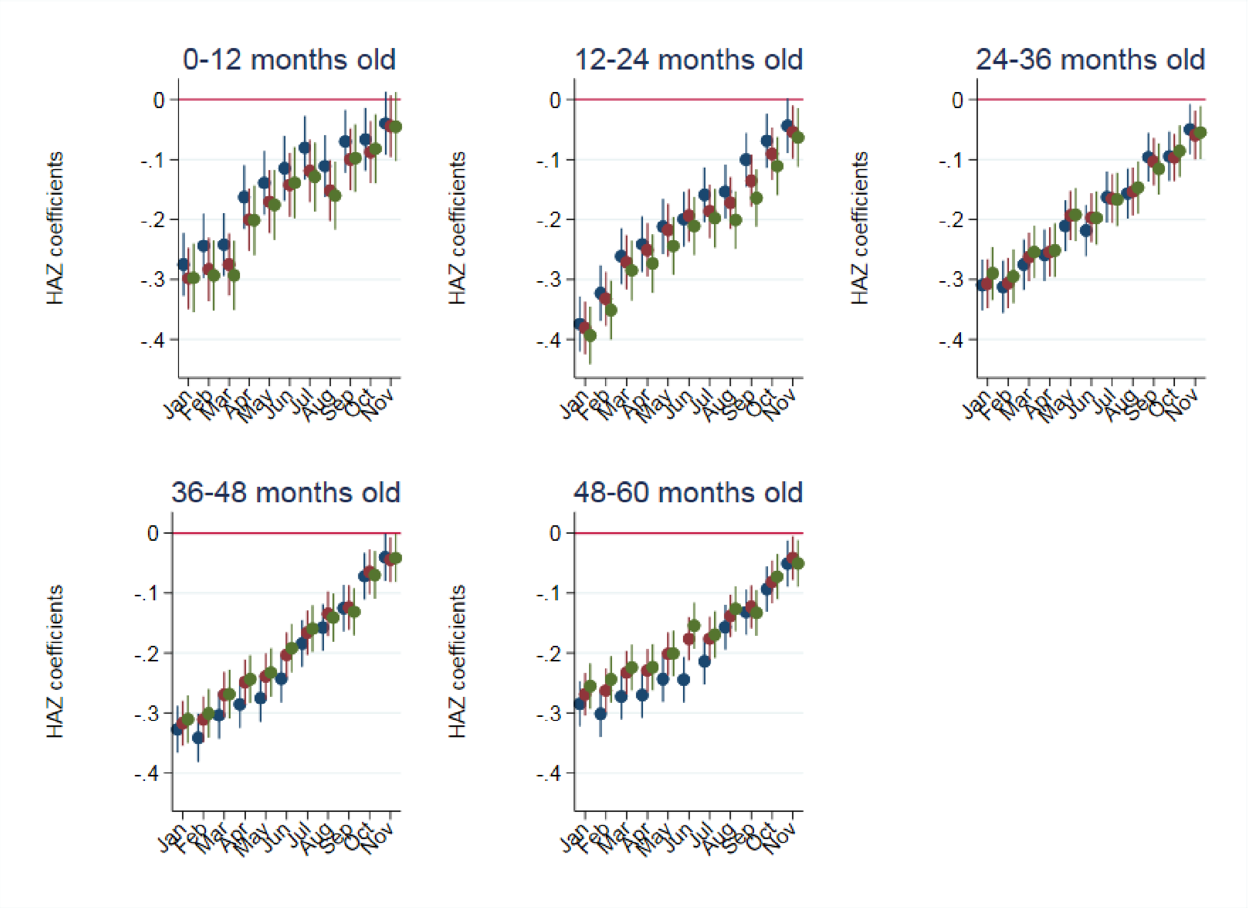

Source: DHS data from 990,231 children in 62 countries, various years.

Note: HAZ = height-for-age z-scores; MOB = month of birth. The graphs present coefficients to MOB dummies from three regressions: raw means (in blue); controlling for child demographics and survey fixed effects (red); and also controlling for parental and household characteristics (green). Regression results are available upon request.

Figure A.10 HAZ by MOB by gender of the child

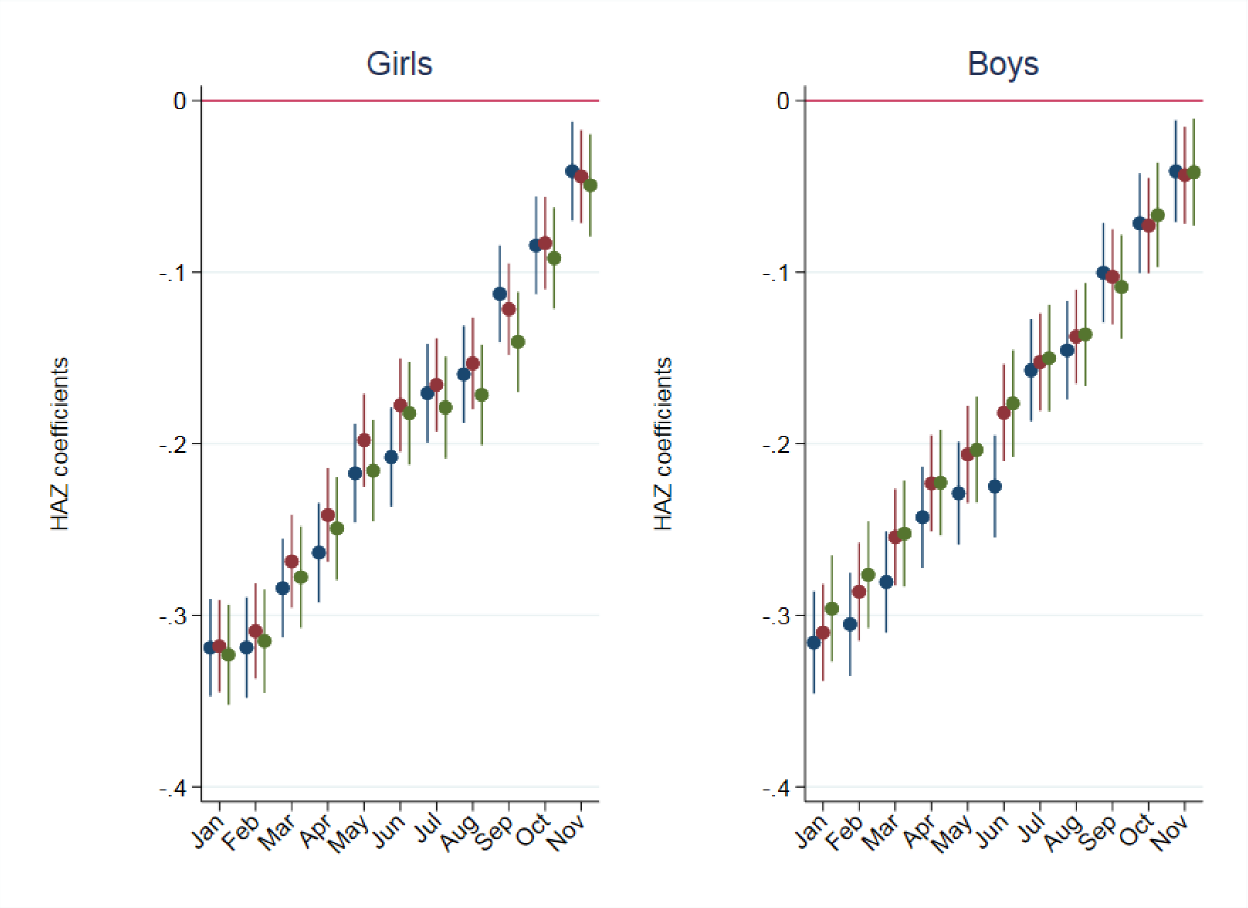

Source: DHS data from 990,231 children in 62 countries.

Note: HAZ = height-for-age z-scores; MOB = month of birth. Fifty-one percent of the children are boys. The graphs present coefficients to MOB dummies from three regressions: raw means (in blue); controlling for child demographics and survey fixed effects (red); and also controlling for parental and household characteristics (green). Regression results are available upon request.

Figure A.11 HAZ by MOB by number of children in the household

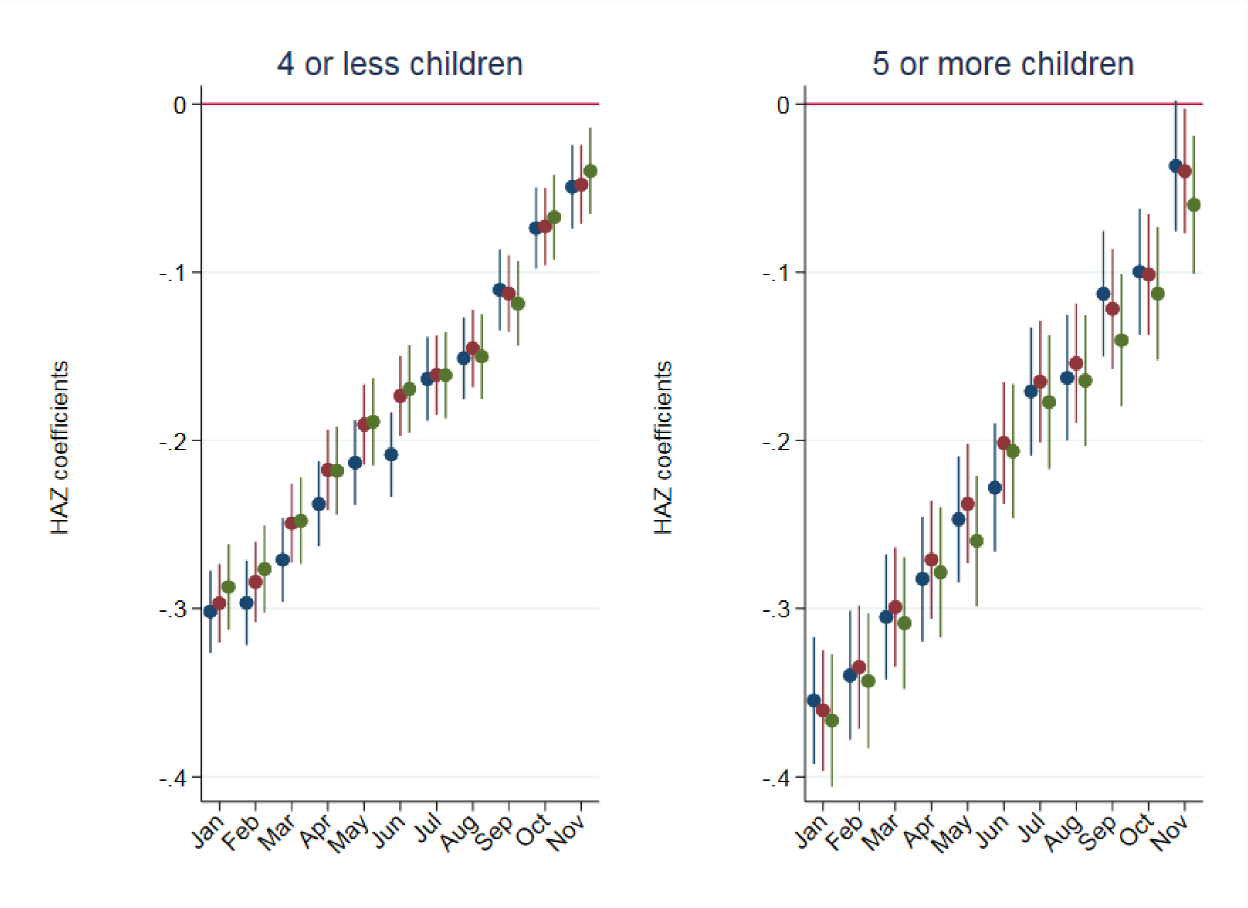

Source: DHS data from 990,231 children in 62 countries.

Note: HAZ = height-for-age z-scores; MOB = month of birth. Sixty-nine percent of children have three or fewer siblings on their mother's side. The graphs present coefficients to MOB dummies from three regressions: raw means (in blue); controlling for child demographics and survey fixed effects (red); and also controlling for parental and household characteristics (green). Regression results are available upon request.

Figure A.12 HAZ by MOB by location of the household

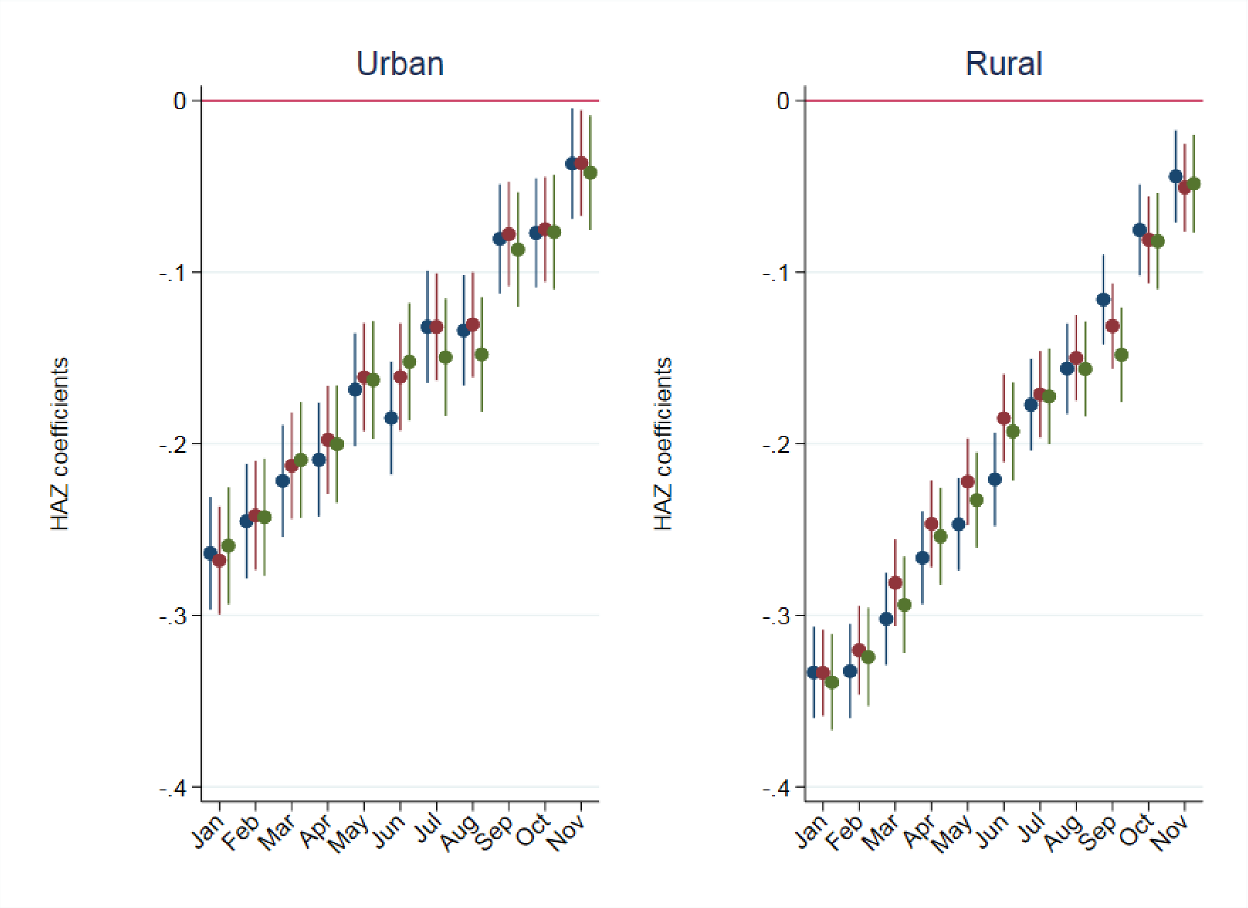

Source: DHS data from 990,231 children in 62 countries.

Note: HAZ = height-for-age z-scores; MOB = month of birth. Thirty-six percent of children live in urban households. The graphs present coefficients to MOB dummies from three regressions: raw means (in blue); controlling for child demographics and survey fixed effects (red); and also controlling for parental and household characteristics (green). Regression results are available upon request.

Figure A.13 HAZ by MOB depending on whether the household has above or below median assets

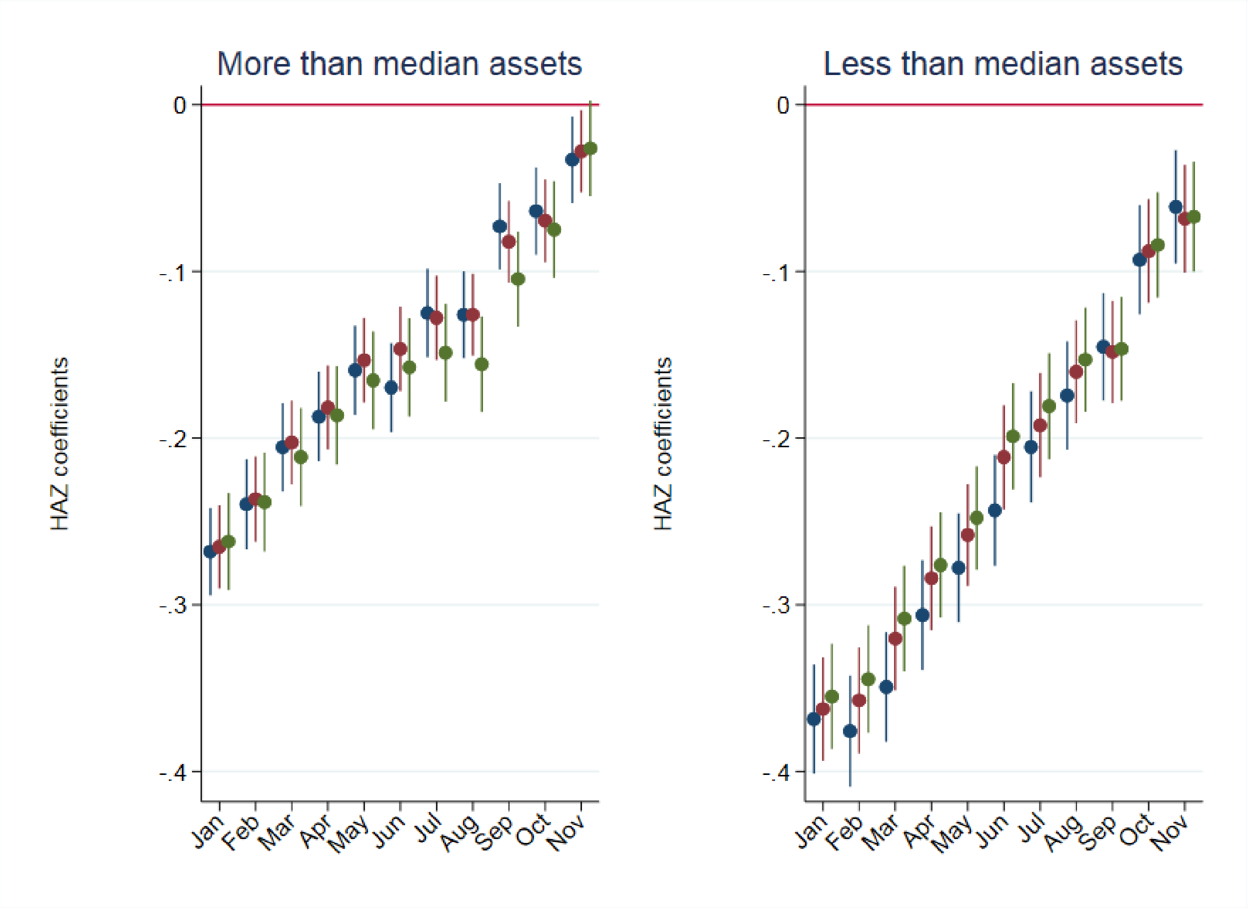

Source: DHS data from 866,450 children in 59 countries.

Note: HAZ = height-for-age z-scores; MOB = month of birth. The graphs present coefficients to MOB dummies from three regressions: raw means (in blue); controlling for child demographics and survey fixed effects (red); and also controlling for parental and household characteristics (green). Regression results are available upon request.

Figure A.14 HAZ by additional months for major regions and selected countries including controls

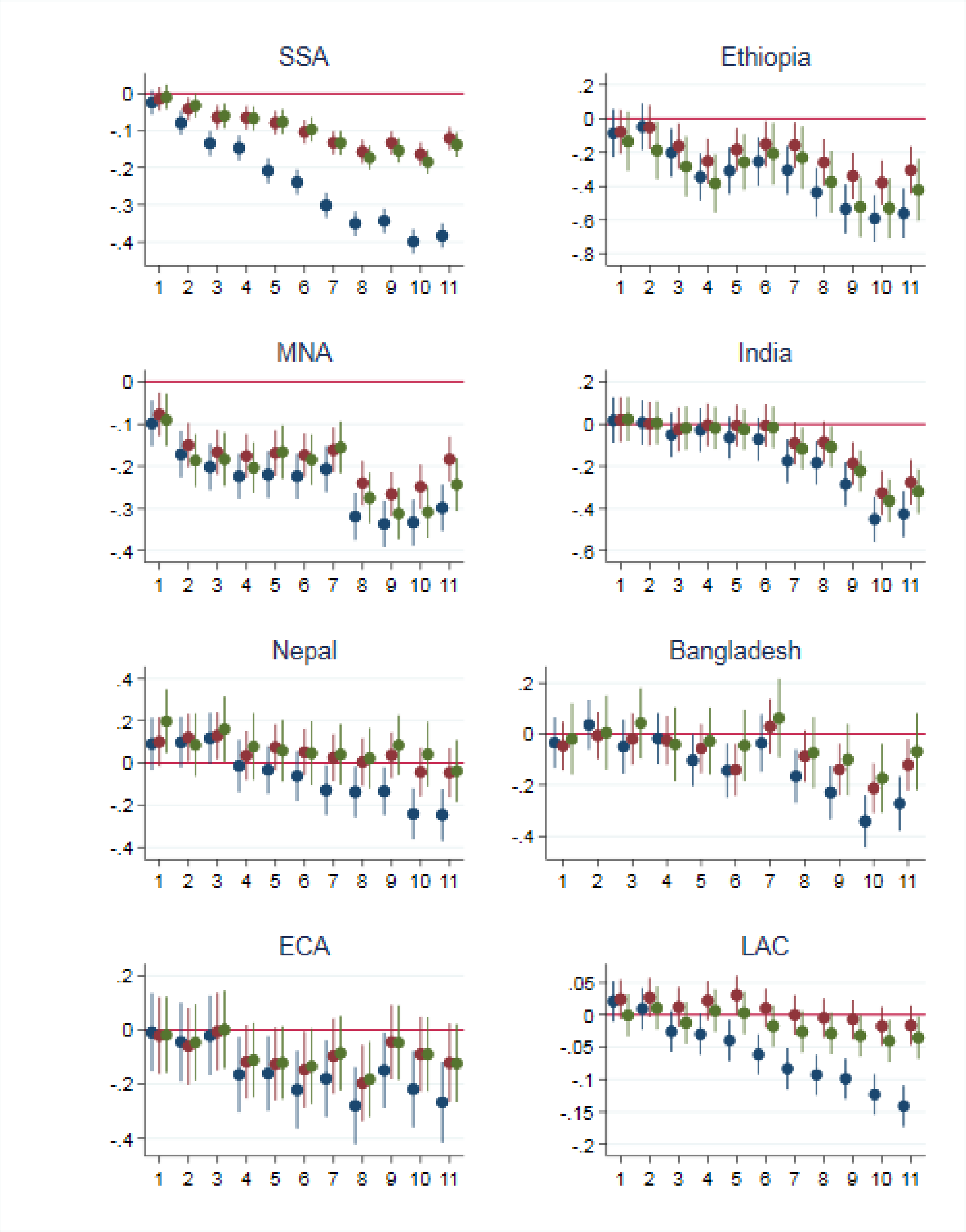

Source: DHS data for 960,012 children from 58 countries, various years.

Note: HAZ = height-for-age z-scores. SSA = Africa south of the Sahara; MNA = Middle East and North Africa; ECA = Eastern Europe and Central Asia; LAC = Latin America and Caribbean. The graphs present coefficients to MOB dummies from three regressions: raw means (in blue); controlling for child demographics and survey fixed effects (red); and also controlling for parental and household characteristics (green). Regression results are available upon request.

Figure A.15 Separate and joint tests for month-of-birth and round age biases in stunting status, with alternative sets of control variables

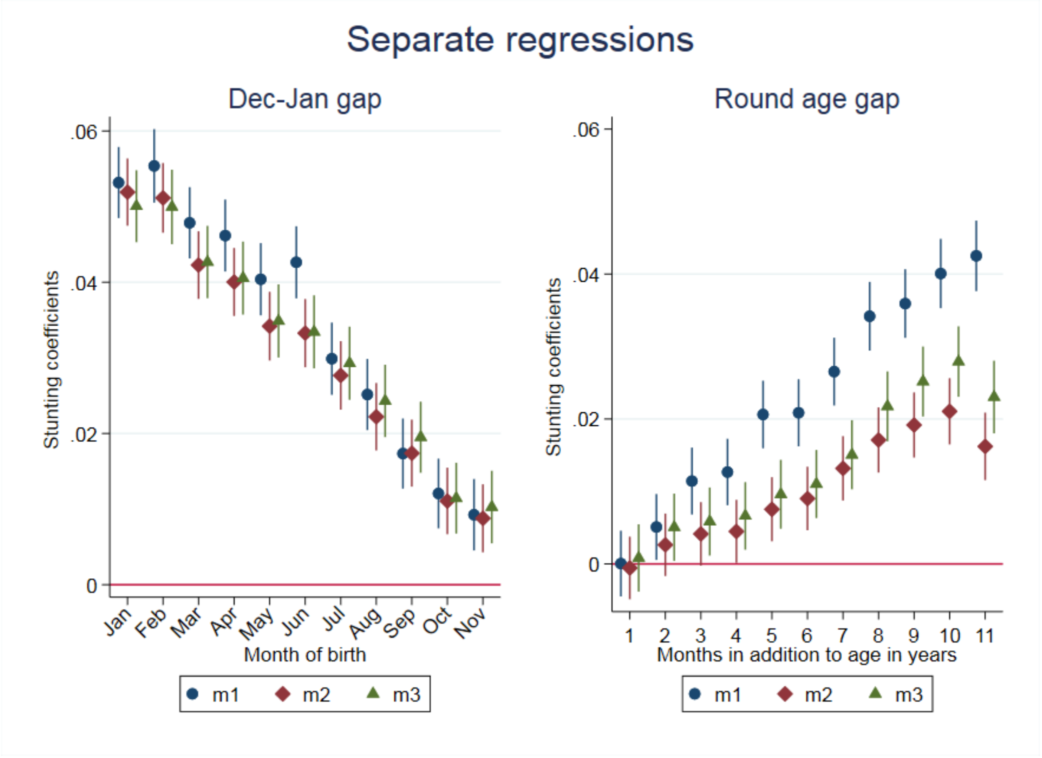

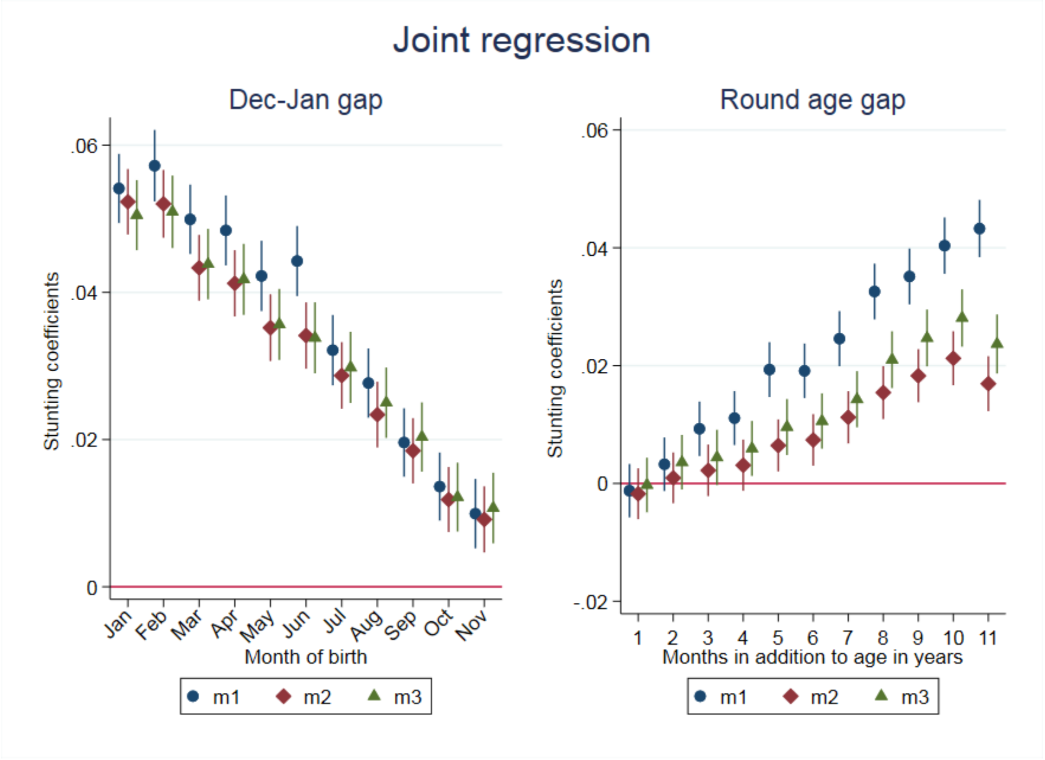

Source: DHS data for 990,231 children from 62 countries, various years.

Note: Results shown are as for Figure 8 in the main text, but for stunting status instead of mean HAZ. Regression results are available upon request.

B: Simulation protocol and additional results

Data-generating process

To simulate the true underlying height data, we implement the following data-generating process. We use Stata 14 for the simulations with the seed 1159 for the random number generator.

1. The observations consist of 100 girls born on each day between January 1, 2010, to December 31, 2015 (219,100 observations in total).
2. Assign a random day of measurement for each observation within the time span January 1, 2015, to December 31, 2015.
3. Calculate the true age (in days) as the difference between the birth date and the day of measurement. This leads to an age range from almost −1 year to 6 years of age. The reason to include children with ages greater than five years is that the measurement error in age may cause children to be included in the sample who are truly too old to be included. We disregard children with negative age (that is, born later than the day of measurement, 18,333 observations). Furthermore, we mirror the increasing attrition with age that we see in the DHS data by dropping a number of observations that increase linearly with age up to 24 percent for those 58 months old as found in the DHS data. Now, the total number of observations is 175,030.
4. Merge the data with age-specific synthetic length/height medians and standard deviations (SDs). These are constructed the following way:
   a) We use World Health Organization (WHO) length/height medians and SDs by age in days for girls as a starting point (WHO MGRSG 2006). These are available up to 1,856 days of age. For older children, WHO provides means and SDs by age in months (de Onis et al. 2007). We make a linear interpolation to obtain means and SDs by age in days for children older than 1,856 days.

   b) The WHO reference data are based on well-nourished children. To illustrate the measurement error in an environment with a plausible amount of stunting, we adjust the medians and SDs to correspond in a smooth way to the empirical pattern from the DHS data.

   c) The height medians are adjusted by changing the growth velocities such that children up to six months grow 7 percent less each day than well-nourished children; children from six months to two years of age grow 21 percent less each day than the growth standards; and children older than two years grow 10 percent less each day than the growth standards. Figure B.1 illustrates how these adjustments calibrate the synthetic mean heights well to the DHS mean heights.

   d) We add 2 to the height SDs to account for overall measurement error and increased dispersion due to variation in nutritional status of the children in the sample. In the DHS data, the SDs of height increase less with age than the WHO SDs, so we multiply the WHO SDs with 0.85 to have the same age gradient in the synthetic data as in the DHS data. Figure B.2 illustrates the SDs of heights by age in days in the DHS data and in the simulated data. We chose SDs that are below the SDs in the DHS data to better fit the overall and severe stunting rates of the simulated data with the stunting rates in the DHS data.

1. Draw heights for each observation from a normal distribution using the synthetic medians and SDs.
2. Calculate the true HAZ based on the simulated data for the children who are younger than 1,826 days (five years). Figure B.3 illustrates how the simulated true HAZ compares to the HAZ in the DHS data.

Figure B.1 Mean height by age (local polynomial smoothing), DHS data and simulated data

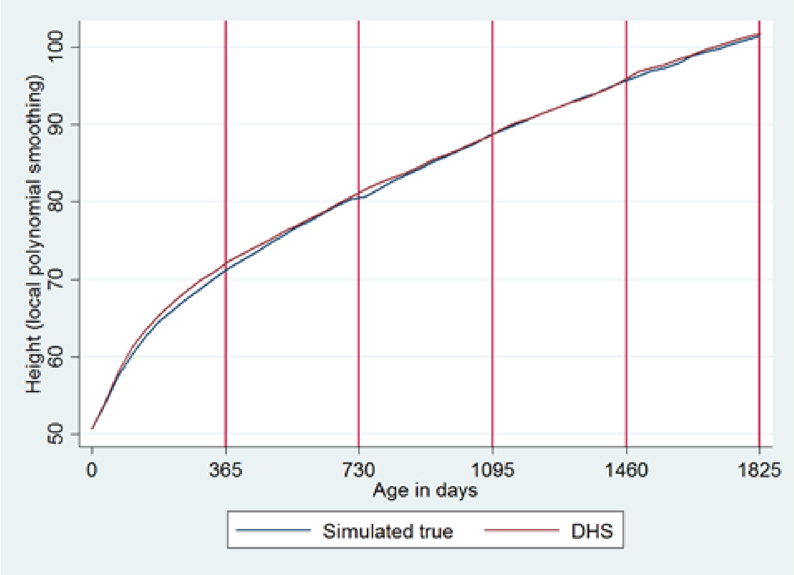

Source: Simulated data and DHS data for 960,012 children from 58 countries, various years.

Note: DHS = Demographic and Health Surveys.

Figure B.2 Standard deviation of height by age (local polynomial smoothing), DHS and simulated data

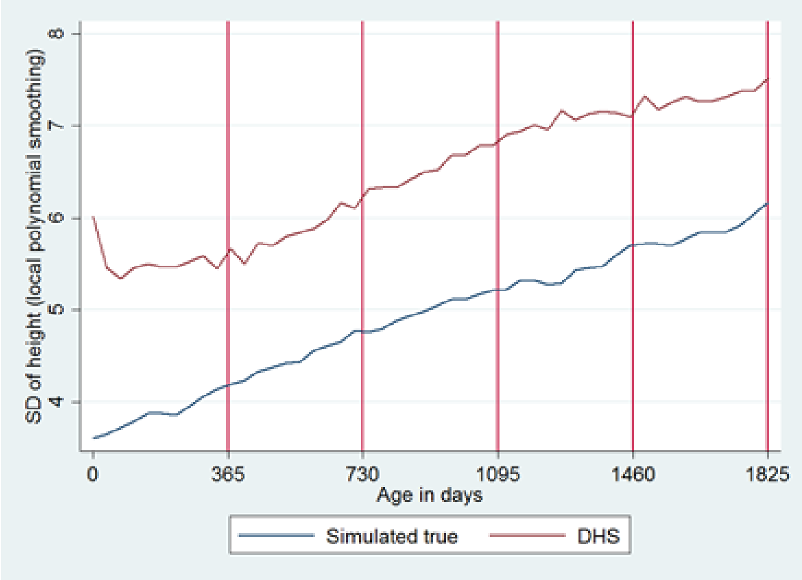

Source: Simulated data and DHS data for 960,012 children from 58 countries, various years.

Note: DHS = Demographic and Health Surveys.

Figure B.3 Mean HAZ by age (local polynomial smoothing), DHS and simulated data

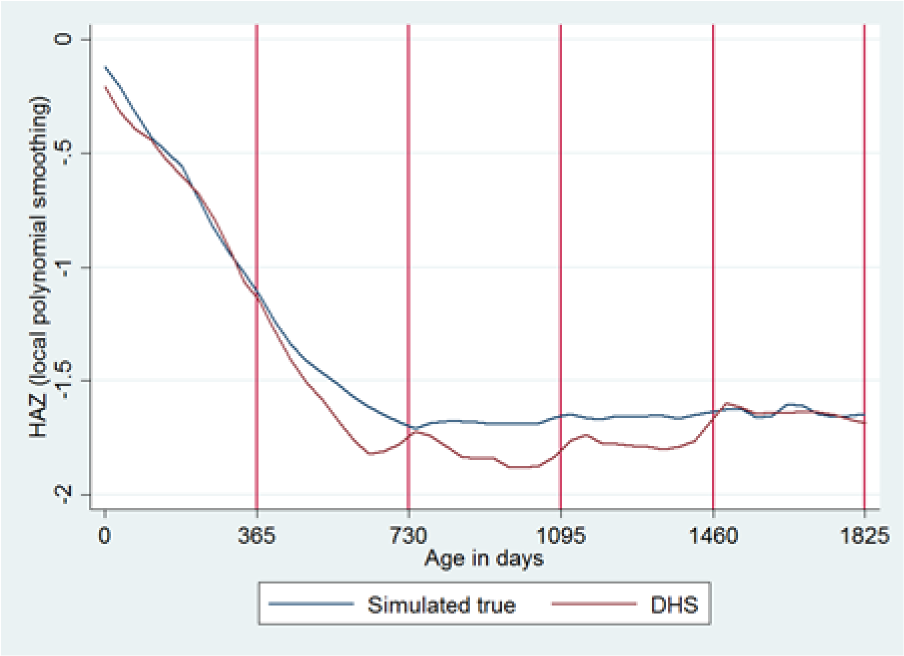

Source: Simulated data and DHS data for 960,012 children from 58 countries, various years.

Note: DHS = Demographic and Health Surveys.

Introducing measurement error: Random month of birth

To illustrate how measurement error in the month of birth can lead to a discontinuity in mean HAZ between December and January and to quantify the impact on stunting rates, we simulate the random month measurement error in the following way:

1. Draw random day and month of birth for each observation from a uniform distribution and calculate reported age based on the random day and month and the true birth year. For children born in 2015, the random month is restricted such that they cannot draw a random month of birth after the month of measurement.
2. Calculate the HAZ with random month of birth error for the children with a reported age below 1,826 days (five years).
3. Show how HAZ with error exhibits qualitatively the same pattern over month of birth as in the DHS data. This is illustrated in Figure B.4.
4. Randomly assign whether a child has measurement error in month of birth or not. We vary the share of children with measurement error to find the share that matches the December–January gap in the simulated mean HAZ with the corresponding gap in the DHS data. This is shown in Figure B.5 and Table B.1.
5. Calculate overall and severe stunting rates for simulated data with varying shares of measurement error in month of birth. These are also included in Table B.1.

Figure B.4 Simulated HAZ by calendar month with and without random months

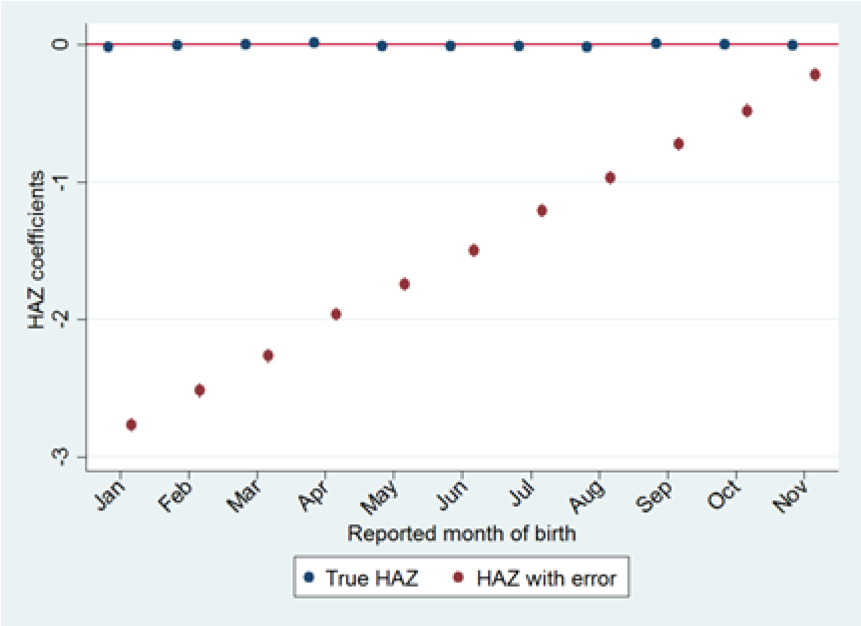

Source: Simulated data and DHS data for 960,012 children from 58 countries, various years.

Note: DHS = Demographic and Health Surveys.

Figure B.5 Simulated HAZ by calendar month at each share of children with random months

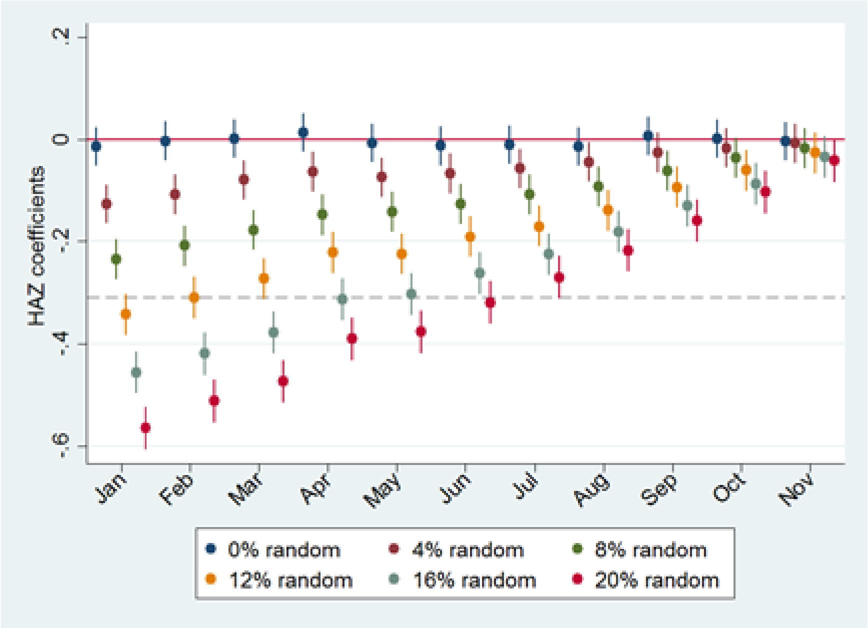

Source: Simulated data.

Note: HAZ = height-for-age z-scores. Dashed line represents December–January gap in DHS data.

Table B.1 Simulated December–January gap and stunting rates by share with random month

Share random	Dec–Jan gap	Overall stunting (<-2)		Severe stunting (<-3)	
DHS	*-0.313*	*0.354*		*0.16*	
		Rate	Difference	Rate	Difference
**0.00**	**-0.014**	**0.352**	**-**	**0.145**	**-**
0.01	-0.038	0.353	+0.001	0.146	+0.001
0.02	-0.064	0.354	+0.002	0.147	+0.002
0.03	-0.095	0.354	+0.002	0.147	+0.002
0.04	-0.126	0.355	+0.003	0.148	+0.003
0.05	-0.155	0.355	+0.003	0.148	+0.003
0.06	-0.181	0.355	+0.003	0.149	+0.004
0.07	-0.209	0.356	+0.004	0.149	+0.004
0.08	-0.235	0.356	+0.004	0.150	+0.005
0.09	-0.259	0.356	+0.004	0.150	+0.005
0.10	-0.287	0.357	+0.005	0.151	+0.006
**0.11**	**-0.320**	**0.357**	**+0.005**	**0.152**	**+0.007**
0.12	-0.343	0.358	+0.006	0.153	+0.008
0.13	-0.368	0.358	+0.006	0.153	+0.008
0.14	-0.402	0.359	+0.007	0.154	+0.009
0.15	-0.428	0.359	+0.007	0.155	+0.010
0.16	-0.456	0.359	+0.007	0.155	+0.010
0.17	-0.483	0.360	+0.008	0.156	+0.011
0.18	-0.511	0.360	+0.008	0.156	+0.011
0.19	-0.539	0.360	+0.008	0.157	+0.012
0.20	-0.565	0.361	+0.009	0.158	+0.013
0.21	-0.592	0.361	+0.009	0.158	+0.013
0.22	-0.620	0.362	+0.010	0.159	+0.014
0.23	-0.649	0.362	+0.010	0.160	+0.015
0.24	-0.680	0.362	+0.010	0.160	+0.015
0.25	-0.707	0.363	+0.011	0.161	+0.016
0.26	-0.759	0.362	+0.010	0.162	+0.017
0.27	-0.789	0.363	+0.011	0.162	+0.017
0.28	-0.811	0.363	+0.011	0.163	+0.018
0.29	-0.841	0.364	+0.012	0.164	+0.019
0.30	-0.870	0.364	+0.012	0.164	+0.019
0.31	-0.899	0.365	+0.013	0.165	+0.020
0.32	-0.932	0.365	+0.013	0.166	+0.021
0.33	-0.957	0.365	+0.013	0.166	+0.021
0.34	-0.985	0.366	+0.014	0.167	+0.022
0.35	-1.013	0.366	+0.014	0.168	+0.023
0.36	-1.038	0.366	+0.014	0.168	+0.023
0.37	-1.073	0.367	+0.015	0.169	+0.024

--continued--					
Share random	Dec–Jan gap	Overall stunting (<-2)		Severe stunting (<-2)	
		Rate	Difference	Rate	Difference
0.38	-1.104	0.367	+0.015	0.169	+0.024
0.39	-1.133	0.367	+0.015	0.170	+0.025
0.40	-1.163	0.368	+0.016	0.171	+0.026
0.41	-1.192	0.368	+0.016	0.171	+0.026
0.42	-1.217	0.369	+0.017	0.172	+0.027
0.43	-1.238	0.369	+0.017	0.172	+0.027
0.44	-1.260	0.369	+0.017	0.173	+0.028
0.45	-1.286	0.370	+0.018	0.174	+0.029
0.46	-1.309	0.370	+0.018	0.174	+0.029
0.47	-1.340	0.371	+0.019	0.175	+0.030
0.48	-1.362	0.371	+0.019	0.175	+0.030
0.49	-1.390	0.371	+0.019	0.176	+0.031
0.50	-1.417	0.372	+0.020	0.176	+0.031

Source: Simulated data.

Note: HAZ = height-for-age z-scores.

Table B.2 Actual December–January gaps, SD of HAZ and estimated share of children with random birthdays for 163 Demographic Health Surveys

Country	Year(s)	Phase	N	Dec-Jan gap	SD of HAZ	Share random
Armenia	2000	4	1539	-0.42	1.47	0.14
Armenia	2005	5	1300	0.06	2.14	0.00
Albania	2008-09	5	1523	-1.08	2.88	0.37
Armenia	2010	6	1406	0.12	2.04	0.00
Azerbaijan	2006	5	2089	-0.06	2.09	0.02
Bangladesh	1999-00	4	5530	-0.14	1.85	0.05
Bangladesh	1996-97	3	5091	-0.42	2.31	0.14
Bangladesh	2004	4	6050	-0.06	1.57	0.02
Bangladesh	2007	5	5399	0.09	1.61	0.00
Bangladesh	2011	6	7867	-0.22	1.80	0.08
Benin	2001	4	4521	-0.08	1.92	0.03
Benin	2006	5	13431	-0.33	2.48	0.11
Benin	2011-12	6	11420	-0.76	3.25	0.26
Bolivia	1998	3	6376	-0.34	2.02	0.12
Bolivia	2003	4	9334	-0.11	1.67	0.04
Bolivia	2008	5	7817	-0.09	1.47	0.03
Brazil	1996	3	4178	-0.09	1.70	0.03
Burkina Faso	1998-99	3	4763	-0.45	2.09	0.15
Burkina Faso	1993	2	4579	-0.43	2.12	0.15
Burkina Faso	2003	4	8795	0.00	2.30	0.00
Burkina Faso	2010	6	6728	-0.48	1.80	0.17
Burundi	2010	6	3494	-0.48	1.53	0.17
Cambodia	2000	4	3776	-0.68	2.07	0.24
Cambodia	2005	5	3682	-0.42	1.69	0.15
Cambodia	2010	6	3807	-0.13	1.75	0.04
Cameroon	2004	4	3336	-0.08	2.05	0.03
Cameroon	2011	6	5188	-0.51	1.92	0.18
Chad	2004	4	4656	-0.29	2.28	0.10
Colombia	1995	3	4561	-0.03	1.27	0.01
Colombia	2000	4	4226	0.03	1.21	0.00
Colombia	2005	5	12480	-0.01	1.21	0.00
Colombia	2010	6	16041	0.01	1.16	0.00
Comoros	2012	6	2711	-0.65	2.90	0.22
Congo, Dem. Rep.	2007	5	3657	-0.48	3.06	0.17
Congo, Dem. Rep.	2013-14	6	8398	-0.72	2.16	0.25
Congo, Rep.	2005	5	4062	-0.40	2.10	0.14
Congo, Rep.	2011-12	6	4531	-0.68	1.60	0.23
Cote d'Ivoire	1998-99	3	1592	-0.37	1.78	0.13
Cote d'Ivoire	2011-12	6	3297	-0.48	2.02	0.16

--continued--						
Country	Year(s)	Phase	N	Dec-Jan gap	SD of HAZ	Share random
Dominican Rep.	1996	3	3810	-0.23	1.57	0.08
Dominican Rep.	2002	4	9447	-0.28	1.51	0.10
Dominican Rep.	2007	5	9489	-0.23	1.54	0.08
Dominican Rep.	2013	6	3238	-0.13	1.37	0.04
Egypt	1992	2	7706	-0.47	1.97	0.16
Egypt	1995	3	10839	-0.70	2.05	0.24
Egypt	2000	4	10721	-0.34	1.85	0.12
Egypt	2003	4	6247	-0.17	1.58	0.06
Egypt	2005	5	13175	-0.21	2.26	0.07
Egypt	2008	5	10469	-0.25	2.60	0.09
Egypt	2014	6	15189	-0.65	2.72	0.23
Ethiopia	2000	4	9066	-0.21	1.98	0.07
Ethiopia	2005	5	4195	-0.55	2.59	0.19
Ethiopia	2011	6	9886	-0.41	2.05	0.14
Gabon	2000	4	3572	-0.45	1.72	0.16
Gabon	2012	6	3490	-0.49	2.02	0.17
Ghana	1998	4	2838	-0.20	1.95	0.07
Ghana	2003	4	3200	-0.20	1.95	0.07
Ghana	2008	5	2526	-0.08	2.36	0.03
Guatemala	1995	3	8794	-0.13	1.61	0.04
Guatemala	1998-99	4	4026	-0.08	1.70	0.03
Guinea	1999	4	4625	-0.62	2.39	0.22
Guinea	2005	5	2754	-0.08	2.09	0.03
Guinea	2012	6	3221	-0.24	2.08	0.09
Guyana	2009	5	1707	-0.76	2.36	0.26
Haiti	2000	4	5628	-0.35	1.59	0.12
Haiti	2005-06	5	2597	-0.24	1.56	0.08
Haiti	2012	6	4042	-0.49	1.54	0.17
Honduras	2005-06	5	9043	-0.12	1.48	0.04
Honduras	2011-12	6	10014	0.04	1.26	0.00
India	2005-06	5	43656	-0.26	2.13	0.09
Jordan	1990	2	6888	-0.05	1.83	0.02
Jordan	1997	3	5675	-0.16	1.37	0.05
Jordan	2002	4	4935	-0.17	1.32	0.06
Jordan	2007	5	4769	-0.47	2.47	0.16
Jordan	2009	6	4429	-0.20	1.47	0.07
Jordan	2012	6	6355	-0.05	1.52	0.02
Kazakhstan	1999	4	580	0.02	1.49	0.00
Kenya	1993	3	5084	-0.50	1.87	0.17
Kenya	1998	3	4815	-0.34	2.23	0.12
Kenya	2003	4	4879	-0.17	1.98	0.06

--continued--						
**Country**	**Year(s)**	**Phase**	**N**	**Dec-Jan gap**	**SD of HAZ**	**Share random**
Kenya	2008-09	5	5340	-0.38	2.19	0.13
Kyrgyz Republic	2012	6	4068	-0.34	1.53	0.12
Lesotho	2004	4	1465	-0.31	2.24	0.11
Lesotho	2009	6	1675	0.33	2.18	0.00
Liberia	2007	5	4566	-0.24	2.37	0.08
Liberia	2013	6	3262	-0.45	1.93	0.15
Madagascar	1992	2	4228	-0.14	1.58	0.05
Madagascar	2003-04	4	4738	-0.47	2.15	0.16
Madagascar	2008-09	5	5534	-0.18	2.57	0.06
Malawi	1992	2	3361	-0.34	1.78	0.12
Malawi	2000	4	9744	-0.42	2.25	0.14
Malawi	2004	4	8686	-0.43	2.34	0.15
Malawi	2010	6	4843	-0.27	2.13	0.10
Maldives	2009	5	2451	-0.10	1.86	0.03
Mali	2006	5	11643	-0.44	2.35	0.15
Mali	2012-13	6	4600	-1.16	2.44	0.41
Moldova	2005	5	1381	-0.41	2.21	0.14
Morocco	1992	2	4652	-0.14	1.80	0.05
Morocco	2003-04	4	5680	-0.32	2.15	0.11
Mozambique	2003	4	8288	-0.52	1.77	0.18
Mozambique	2011	6	9721	-0.46	1.93	0.16
Namibia	1992	2	2768	-0.05	1.87	0.02
Namibia	2000	4	3038	-0.40	1.84	0.14
Namibia	2006-07	5	3846	-0.30	2.01	0.10
Namibia	2013	6	1880	-0.49	2.39	0.17
Nepal	2001	4	6252	-0.07	1.42	0.02
Nepal	2006	5	5283	0.05	1.39	0.00
Nepal	2011	6	2360	-0.18	1.55	0.06
Nicaragua	1998	3	7168	-0.39	1.98	0.14
Nicaragua	2001	4	6103	0.18	1.82	0.00
Niger	2006	5	3869	-0.26	2.09	0.09
Niger	2012	6	5153	-0.25	2.53	0.09
Nigeria	1990	2	6150	-0.62	2.27	0.22
Nigeria	2003	4	4793	-0.84	2.63	0.29
Nigeria	2008	5	23123	-0.90	3.44	0.31
Nigeria	2013	6	26827	-0.69	2.74	0.24
Pakistan	1990-91	2	4681	-0.26	2.36	0.09
Pakistan	2012-13	6	3640	-0.38	3.12	0.13
Paraguay	1990	2	3682	-0.14	1.42	0.05
Peru	1991-92	2	7870	-0.30	1.63	0.10
Peru	1996	3	15259	-0.29	1.72	0.10

--continued--						
**Country**	**Year(s)**	**Phase**	**N**	**Dec-Jan gap**	**SD of HAZ**	**Share random**
Peru	2000	4	11795	-0.23	1.52	0.08
Peru	2003-08	5	10494	-0.03	1.26	0.01
Peru	2009	5	9406	-0.09	1.19	0.03
Peru	2010	6	8804	0.16	1.15	0.00
Peru	2011	6	8753	0.03	1.11	0.00
Peru	2012	6	8893	-0.17	1.09	0.06
Rwanda	1992	2	4415	-0.17	1.72	0.06
Rwanda	2000	4	6380	-0.51	2.17	0.18
Rwanda	2005	5	3786	-0.06	1.95	0.02
Rwanda	2010	6	4122	-0.15	1.47	0.05
Sao Tome & Principe	2008-09	5	1706	-0.77	2.80	0.27
Senegal	1992-93	2	4665	-0.21	1.90	0.07
Senegal	2005	4	2936	-0.10	1.79	0.03
Senegal	2010-11	6	3929	-0.44	2.41	0.15
Senegal	2012-13	6	6070	-0.17	1.80	0.06
Senegal	2014	7	6110	-0.01	1.65	0.00
Sierra Leone	2008	5	2285	-0.98	2.60	0.34
Sierra Leone	2013	6	4729	-0.76	3.44	0.26
Swaziland	2006-07	5	2106	-0.12	1.80	0.04
Tajikistan	2012	6	4767	-0.50	2.05	0.17
Tanzania	1991-92	2	6742	-0.23	1.98	0.08
Tanzania	1996	3	5576	-0.29	2.01	0.10
Tanzania	1999	4	2584	-0.10	1.62	0.04
Tanzania	2004-05	4	7303	-0.24	1.54	0.09
Tanzania	2010	6	6955	-0.19	1.75	0.07
Timor-Leste	2009-10	6	8182	-1.03	2.49	0.36
Turkey	1993	3	3187	-0.29	1.62	0.10
Turkey	1998	4	2845	-0.24	1.58	0.08
Turkey	2003	4	4074	0.04	1.52	0.00
Uganda	2000-01	4	5270	-0.18	1.75	0.06
Uganda	2006	5	2421	-0.28	1.89	0.10
Uganda	2011	6	2108	-0.12	1.71	0.04
Yemen	1991-92	2	2959	-1.04	1.69	0.36
Yemen	2013	6	14287	-0.56	2.00	0.20
Zambia	1992	2	5084	-0.36	1.73	0.13
Zambia	1996	3	5678	-0.34	1.80	0.12
Zambia	2001-02	4	5643	-0.26	1.97	0.09
Zambia	2007	5	5391	-0.38	2.22	0.13
Zambia	2013-14	6	11799	-0.46	1.88	0.16
Zimbabwe	1999	4	2848	-0.14	2.45	0.05
Zimbabwe	2005-06	5	4211	0.21	2.29	0.00

--continued--						
**Country**	**Year(s)**	**Phase**	**N**	**Dec-Jan gap**	**SD of HAZ**	**Share random**
Zimbabwe	2010-11	6	4412	-0.36	1.70	0.12

Notes: Phase refers to the DHS phase. N refers to the number of observations; Dec-Jan gap refers to the Dec-Jan Gap in HAZ scores based on MOB; SD refers to the standard deviation of HAZ; and share random refers to the estimated share of the children who may have a purely random MOB.
